# Response Regimes in On-Chip THz Spectroscopy

**DOI:** 10.1021/acsphotonics.6c00409

**Published:** 2026-07-03

**Authors:** Gunda Kipp, Marios H. Michael, Alexander M. Potts, Dorothee Herrmann, Toru Matsuyama, Guido Meier, Matthew W. Day, Hope M. Bretscher, James W. McIver

**Affiliations:** † 375070 Max Planck Institute for the Structure and Dynamics of Matter, Luruper Chaussee 149, 22761 Hamburg, Germany; ‡ Center for Free-Electron Laser Science (CFEL), Luruper Chaussee 149, 22761 Hamburg, Germany; § 28269 Max Planck Institute for the Physics of Complex Systems, Nöthnitzer Straße 38, 01187 Dresden, Germany; ∥ Department of Physics, 5798 Columbia University, 538 West 120th Street, New York, New York 10027, United States

**Keywords:** Plasmonic self-cavity, finite-momentum THz responses, near-field spectroscopy, 2D materials, Phantom-Drude response, inhomogeneous linewidth broadening, gate hybridization

## Abstract

On-chip THz spectroscopy enables quantitative measurements of the optical conductivity of subwavelength 2D materials by tightly confining THz fields in metallic transmission line structures interfaced to the material. However, because the probed structures are smaller than the THz wavelength, finite-size and environmental effects can strongly influence the measured response. Here, we identify the conditions under which the near-field conductivity of a metallic sample exhibits a genuine Drude response and when finite-size and environmental effects must be considered. We introduce and characterize an unexplored regime, the Phantom-Drude response, which mimics Drude behavior but instead originates from the superposition of multiple finite-momentum plasmonic resonances. If unrecognized, this regime can lead to the misinterpretation of intrinsic material properties. We systematically show how the Phantom-Drude response can emerge and demonstrate its sensitivity to sample dimensions, transmission line geometry, material shape, and gate properties, providing practical guidelines for avoiding this regime in on-chip THz measurements.

## Graphical abstract

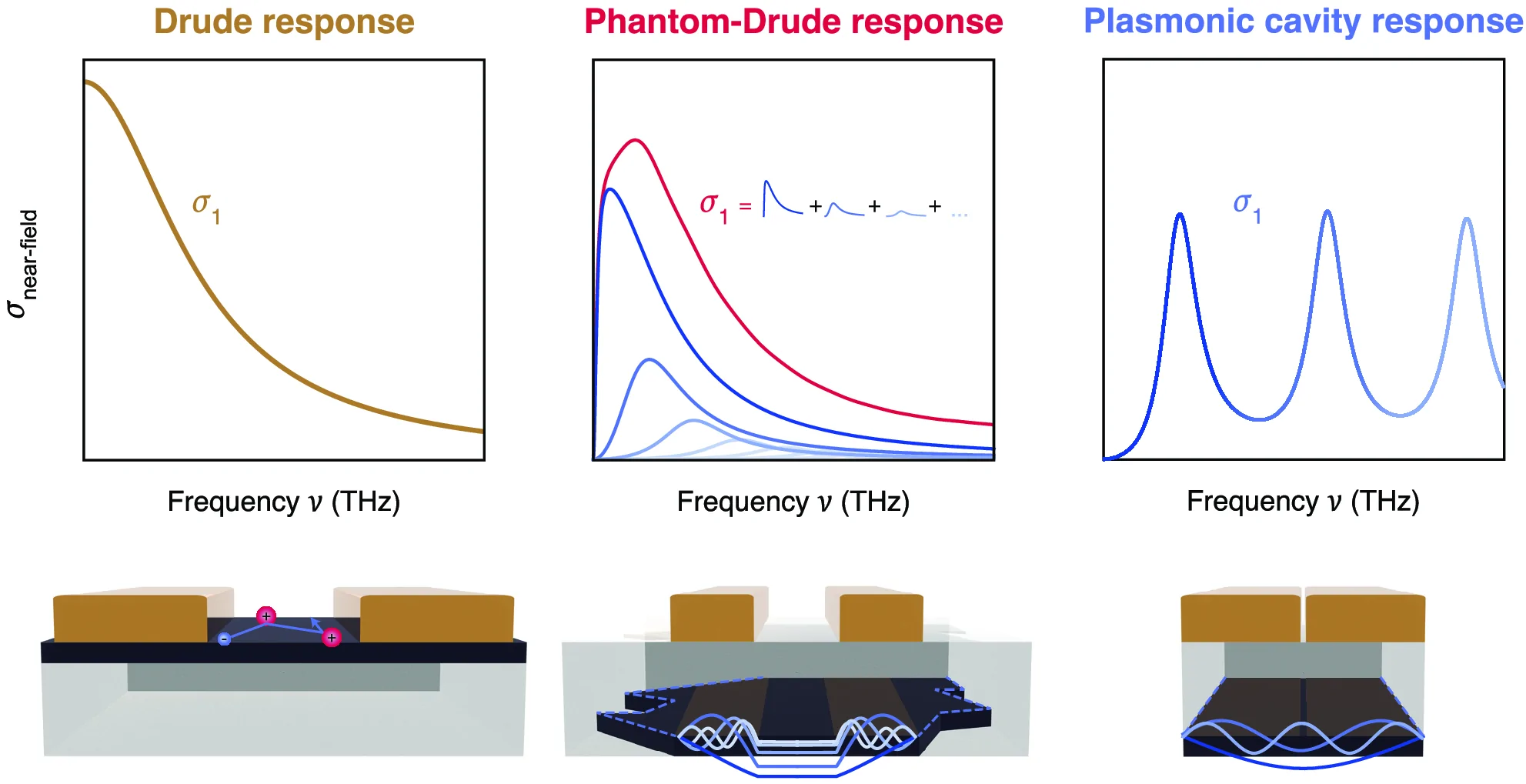

## Introduction

Two-dimensional (2D) materials and van der Waals (vdW) heterostructures host a variety of emergent, gate-tunable phenomena.^1–7^ Many of these, such as superconductivity, correlated insulating states, and strange-metal phases, arise at μeV–meV energy scales,^8–13^ corresponding to terahertz (THz) frequencies. While conventional far-field THz techniques can access these frequencies, they are diffraction-limited and cannot resolve micron-scale samples smaller than the THz wavelength. This limitation has spurred the development of near-field THz techniques capable of probing subwavelength structures.^14–20^ Crucially, signals from subwavelength samples can differ qualitatively from far-field measurements of large-area samples, as finite-size and environmental effects dominate at small scales.^21^

Of the available near-field methods, on-chip THz spectroscopy^22–29^ is uniquely suited to probe the influence of subwavelength THz effects on a sample’s response because there is an analytical mapping between the near-field signal and the intrinsic conductivity.^30^ In this approach, vdW heterostructures are integrated into ultrafast optoelectronic circuits^31–43^ that confine THz fields using lithographically defined metallic transmission lines (Figure 1a). Femtosecond-driven photoconductive switches launch and detect THz signals, allowing the extraction of the complex near-field conductivity, σ_near‑field_, which directly reflects both the intrinsic electronic response and the local dielectric environment.^22^

**Figure 1:**
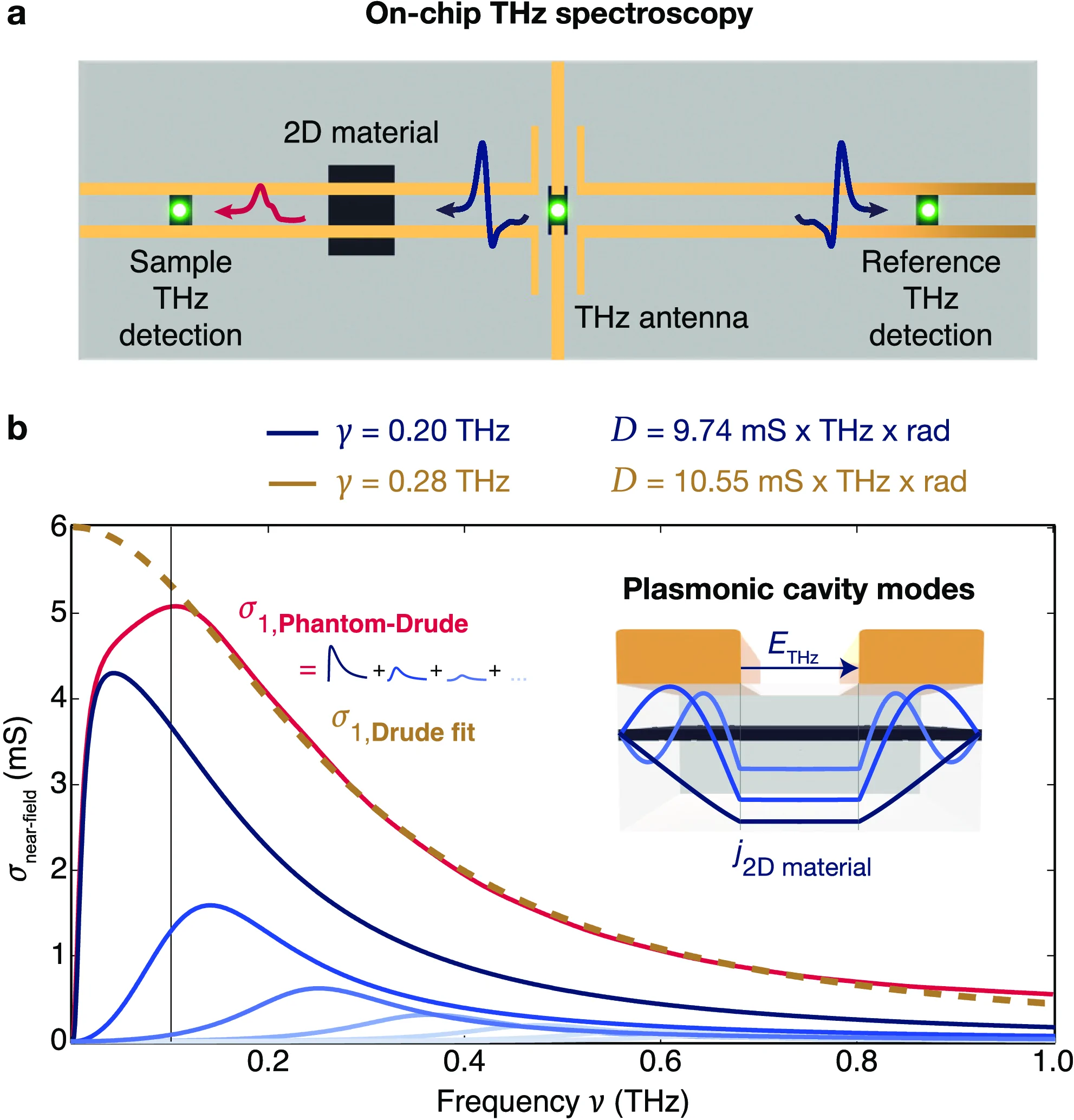
Probing the near-field optical conductivity of 2D materials with on-chip THz spectroscopy: (a) Schematic of the on-chip THz circuit architecture: two identical THz pulses (blue) propagate along symmetric transmission lines; one interacts with a 2D material and is detected on the left (red), while the other serves as a reference on the right. Comparing both signals yields the material’s near-field optical conductivity. (b) 2D materials embedded in on-chip THz spectroscopy circuitry form plasmonic self-cavities, featuring one or more resonances in the near-field optical conductivity spectrum.^22^ These resonances arise from finite-momentum standing waves of current density spanning the sample, as illustrated for the three lowest frequency modes of σ_near‑field_ (blue). When the resonance linewidth γ and the frequency resolution are broader than the mode spacing, overlapping resonances merge into a broad spectrum that mimics a Drude response (red), despite being fundamentally different. A Drude fit to the red spectrum (gold dashed curve) yields misleading parameters: the extracted scattering rate γ and Drude weight *D* (gold) differ significantly from the intrinsic material values (blue). We discuss the origin of this effect and mitigation strategies in this work. Sample parameters for calculations: *W*_0_ = 0 μm (2D material width exceeding the metal strips), *W*_1_ = 10 μm (width of each metal trace), *W*_2_ = 10 μm (width of the gap between strips), *d*_gold_ = 285 nm (gold thickness), *d*_hBN_ = 20 nm (hBN thickness), *D* = 9.74 mS × THz × rad (Drude weight of the 2D material), and γ = 0.2 THz (scattering rate of the 2D material).

Previous on-chip THz spectroscopy works have interpreted the near-field optical conductivity of metallic samples in one of two ways. One group of studies attributed the spectral features to a Drude response,^24–26,44^ where zero-momentum charge transport is governed by electron scattering and dissipation. Another set of studies reported finite-momentum plasmonic resonances,^22,23,45,46^ arising from standing wave patterns of current density that are reflected off of both the edges of the transmission lines and the edges of the THz subwavelength 2D material. Here, the material effectively forms a plasmonic *self-cavity* for THz light^30^ (see inset of Figure 1b). The coexistence of these two interpretations in the literature highlights the importance of identifying not only the conditions under which each applies but also the nature of the transition between them, to ensure the accurate extraction of material parameters.

In this work, we analytically examine the factors that give rise to Drude or cavity responses and reveal an intermediate regime, the *Phantom-Drude* response. This regime has not been explicitly distinguished in prior analyses and, if unrecognized, can lead to erroneous interpretations of the near-field conductivity. While it is known that time-domain reflections or poor choice of window functions can obscure spectral features from collective modes,^47^ we show that the Phantom-Drude response arises from a fundamentally different mechanism: the superposition of multiple, inhomogeneously broadened finite-momentum plasmonic collective modes. As illustrated in Figure 1b, the overlap of several distinct plasmon resonances (blue) can produce a broad, featureless spectrum (red). Such a spectrum appears Drude-like when the measurement bandwidth is limited to the range of 0.1 THz to 1 THz, as is often the case experimentally. However, fitting such a spectrum with a Drude model (gold dashed line) leads to the extraction of material parameters that can be 10% to 400% off from their intrinsic values.

We analyze three mechanisms underlying the Phantom-Drude regime, arising from the transmission line design, sample geometry, and choice of gate materials, and provide practical design guidelines to mitigate them. These insights enable future on-chip THz spectroscopy studies to reliably probe the intrinsic properties of the vdW heterostructures.

## Mechanism 1: Reflection of Current Set by Transmission Line Design

The analytical framework for on-chip THz spectroscopy^22,30^ calculates the effective near-field conductivity for structures where an insulating layer separates the 2D material from the transmission line, capturing how the response is shaped by both the sample geometry and the local dielectric environment. The theory shows that the THz field, largely confined between the transmission line metal strips, drives in-plane currents in the 2D metallic layers perpendicular to the pulse propagation direction (see the inset of Figure 1b). At screened regions beneath the metal traces (*W*_1_), the unscreened gap between the traces (*W*_2_), and the material regions extending beyond the width of the transmission line (*W*_0_), these currents undergo partial reflection and transmission (see Figure 2a) due to the different dispersion relations of screened and unscreened 2D plasmons.

**Figure 2:**
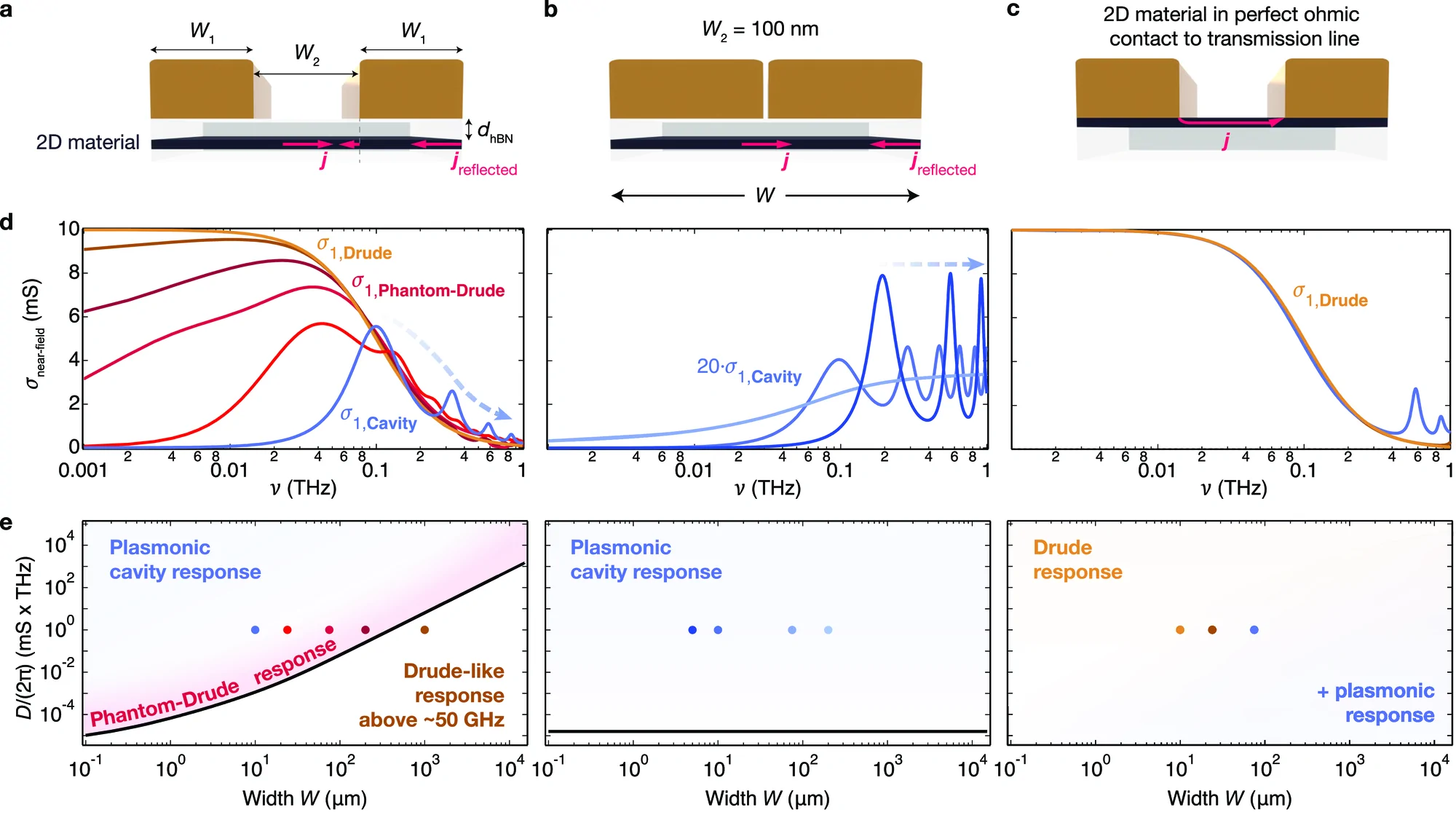
Impact of transmission line design and material conductivity on the measured response: Three transmission line designs are investigated. (a) In the first design, a 2D material is electrically insulated from the transmission line by a hBN layer of thickness *d*_hBN_ = 20 nm. The structure is embedded within a transmission line of total width *W* = 2*W*_1_ + *W*_2_ with *W*_1_ = *W*_2_. No material is extending beyond the transmission line (*W*_0_ = 0, see SI). (b) In the second design, the transmission line has a small gap of width *W*_2_ = 100 nm that is kept constant while tuning the overall width *W*. (c) In the third design, the hBN spacer is removed, and the 2D material is placed in perfect ohmic contact to the transmission line of total width *W* = 2*W*_1_ + *W*_2_ with *W*_1_ = *W*_2_. (d) Displays the real parts of the near-field optical conductivity as a function of frequency corresponding to the markers in panel (e). (e) The nature of the response (whether plasmonic cavity, Phantom-Drude, or Drude) is calculated as a function of *W* and the intrinsic Drude weight *D* of the material, assuming a fixed scattering rate of 0.1 THz, for each of the three designs illustrated in (a–c). For the design shown in (a), any of the three responses (plasmonic cavity, Phantom-Drude, or Drude) can be observed depending on *W* and *D*. In contrast, the design in (b) permits only cavity responses or broad spectral features that do not resemble a Drude response. The design in (c), featuring an ohmic contact, consistently produces a Drude response regardless of *W* or *D*. Additionally, plasmonic resonances can appear on top of the Drude response for large or low-conductive samples due to the formation of unscreened plasmons in the transmission line gap (see SI). The black curves in the left (data adapted from Figure 2d of ref 22) and center panel mark parameter sets (*D*, *W*) for which the real part of the near-field optical conductivity, measured via on-chip THz spectroscopy, deviates less than 10% from the intrinsic 2D conductivity at 10 GHz. Notably, the Phantom-Drude regime (red-shaded area in (e)) expands with increasing linewidth, while the black curve remains fixed. Note that for design (c), full 3D electromagnetic simulations were used in place of the analytical theory (see SI).

In the screened regions, the currents form 2D plasmons with resonance frequencies $\omega_{1}=\sqrt{{\mathrm{Dq}}_{1}^{2}d/\epsilon_{0}\epsilon_{1}}$ , while in the unscreened regions the 2D plasmons obtain higher resonance frequencies $\omega_{0,2}=\sqrt{{\mathrm{Dq}}_{0,2}/\epsilon_{0}\left(\epsilon_{1}+\epsilon_{2}\right)}$ , and ω = 2*πν*.^48,49^ Here, *D* is the Drude weight, defining σ_Drude_ = *D*/(2π­(γ – *iν*)) with scattering rate γ, ϵ_0_ is the vacuum permittivity, *d* and ϵ_1_ denote the thickness and dielectric constant of the insulating layer, and ϵ_2_ the substrate dielectric. The 2D plasmon momenta are set by the widths of the corresponding regions (*q*_0,1_ ≈ *a*π/(2*W*_0,1_) and *q*_2_ ≈ *a*π/*W*_2_, with *a* = 1, 3, 5, ···). Each permitted momentum, and therefore each excited plasmon wavelength (see inset of Figure 1b), selects a distinct point on the dispersion relation, giving rise to a corresponding resonance.

Accounting for all boundary conditions (refs 22,30 ), the overall effective near-field conductivity of the entire heterostructure is given by

1 $$\sigma_{\mathrm{near}‐\mathrm{field}}=\sigma_{\mathrm{Drude}}\left(1+F\right)$$

with the feedback factor for *W*_0_ = 0 (see SI for *W*_0_ ≠ 0)^30^

2 $$F=\frac{q_{2}\mathrm{sinc}\left(q_{2}W_{2}/2\right)}{-q_{2}\cos\left(q_{2}W_{2}/2\right)+q_{1}\sin\left(q_{2}W_{2}/2\right)\tan\left(q_{1}W_{1}\right)}$$

Here, the finite momenta of the screened (*q*_1_) and unscreened (*q*_2_) 2D plasmonic modes with scattering rate γ are given by $q_{1}=\sqrt{\epsilon_{0}\epsilon_{1}\omega \left(\omega +2\pi i\gamma \right)/\left(\mathrm{dD}\right)}$ and *q*_2_ = ϵ_0_(ϵ_1_ + ϵ_2_)­ω­(ω + 2*πiγ*)/*D*. As shown in eqs 1 and 2, the feedback factor *F* alone determines whether the effective near-field conductivity follows a Drude response (*F* ≈ 0) or exhibits self-cavity effects with well-defined plasmonic resonances (*F* ≠ 0). Since *F* depends on the 2D material’s Drude weight *D*, the sample geometry (*W*_1_, *W*_2_, *d*, and *W*_0_), the surrounding dielectric environment (ϵ_1_ and ϵ_2_), and the scattering rate γ, the type of response observed in on-chip THz spectroscopy is entirely determined by the sample’s conductivity, dimensions, and transmission line design.

We therefore theoretically examine three distinct transmission line designs to highlight how they govern the near-field response (see Figure 2): (a) a transmission line with equal metal trace and gap widths (*W*_1_ = *W*_2_), where an insulating hBN layer electrically isolates the 2D material from the transmission line, (b) a design with a small gap width (*W*_2_ ≪ *W*_1_), also incorporating an hBN spacer, and (c) a configuration identical to (a) but without an hBN layer, allowing the 2D material to form an ohmic contact with the transmission line. We then calculated the near-field optical conductivity for a metallic sample with Drude weight *D* = 6.28 mS × THz × rad and scattering rate γ = 0.1 THz. These values are representative of a thin graphite or gated graphene flake, providing an illustrative example of materials that display a Drude response in the far-field but exhibit plasmonic behavior in the near-field.^22,50^ We evaluated the response over a range of sample widths *W* (see Figure 2d) and mapped the resulting response types as a function of both *D* and the total sample width *W* for each transmission line geometry (see Figure 2e). The black contours in Figure 2e indicate the boundary within which the near-field optical conductivity deviates by less than 10% from the intrinsic Drude conductivity at 10 GHz, corresponding to |*F*| < 0.1 at 10 GHz and *F* ≈ 0 at frequencies within the typical experimental bandwidth (see Figure S18 of ref 22). We chose this benchmark because a Drude response is recovered above 100 GHz and scattering rates and Drude weights can be accurately extracted from a near-field spectrum.

For design (a), plasmonic resonances appear prominently in the near-field optical conductivity of a sample with Drude weight *D* = 6.28 mS × THz × rad and a narrow total width *W* = 2*W*_1_ + *W*_2_ = 10 μm (blue trace, Figure 2d). As *W* increases, the resonances shift to lower frequencies (red traces), and their superposition produces a Phantom-Drude response that persists up to *W* = 200 μm for this Drude weight. Only for widths approaching *W* ∼ 1 mm (dark golden trace) does the spectrum begin to resemble a Drude response above ∼50 GHz. Because current on-chip THz circuits typically lack reliable sensitivity below approximately 50 to 100 GHz due to time-domain reflections, such a spectrum could be fitted with a Drude model to reliably extract intrinsic material parameters.

However, widths approaching 1 mm remain challenging to realize in vdW heterostructures. A Drude-like response at experimentally accessible dimensions can instead be achieved only when the Drude weight is sufficiently low, as both increasing *W* and decreasing *D* shift the plasmonic resonances to lower frequencies (Figure 2e, left). Even in this case, however, care must be taken to confirm that the observed spectrum reflects a true Drude response rather than a Phantom-Drude one (see the SI).

One key aspect for the appearance of the Phantom-Drude response in design (a) is the momentum-dependent amplitude decay of the plasmonic self-cavity modes: lower-frequency modes exhibit higher amplitudes, while higher-frequency modes decay in amplitude, as illustrated with the blue dashed arrow in the left panel of Figure 2d. As the self-cavity modes shift to lower frequencies with an increasing sample width or decreasing Drude weight, their superposition can mimic a Drude spectrum, where conductivity decreases with frequency.

In contrast, the 2D material in design (b) is predominantly screened due to the narrow transmission line gap (*W*_2_ ≪ *W*_1_). As a result, the self-cavity modes exhibit the characteristics of screened 2D plasmons with uniform amplitudes across all excited momenta (see dashed blue arrow in center panel of Figure 2d). Consequently, the resulting superposition of plasmonic modes does not mimic a Drude response, even as the sample width *W* is increased or the Drude weight *D* is decreased (see Figure 2d,e, center panels). We note that the model does not account for Landau damping of high-momentum modes or other momentum-dependent scattering mechanisms, which could suppress or broaden higher-momentum excitations.

While we focus here on two representative cases, design (a) with *W*_1_ = *W*_2_ and *W*_0_ = 0, and design (b) where *W*_2_ ≪ *W*_1_ and *W*_0_ = 0, we have also simulated a wide range of geometries with *W*_2_ ≠ *W*_1_ and *W*_0_ ≠ 0 (see the SI). These simulations reveal that the Phantom-Drude response persists in all cases, disappearing only in the limit *W*_2_ ≪ *W*_1_ and *W*_0_ = 0, corresponding to design (b).

Finally, when a 2D material is placed in direct ohmic contact with the transmission line, as shown in design (c) of Figure 2c, a true Drude response (*F* = 0 at all frequencies) is observed regardless of the sample width or Drude weight (see Figure 2d,e, right panels). This is because no plasmons form beneath the metal of the transmission line and a DC current can flow at zero frequency. However, plasmons can form in the unscreened *W*_2_ region, adding resonant features on top of the Drude background. Notably, such plasmonic resonances fall within the experimental bandwidth only for sufficiently large samples or for materials with low conductivity (see the SI).

Based on these three design characterizations, the potential for a Phantom-Drude response arises predominantly in a transmission line geometry where $W_{2}\ll ̷W_{1}$ and $W_{0}\ll ̷W_{1}$ , so that the plasmons that form in the unscreened regions generate self-cavity resonances with momentum-dependent amplitudes. This also requires that the 2D material is electrically isolated from the transmission line by an insulating layer such as hBN (design (a)). While design (b) can also produce a broad spectrum, this design has a clear cavity response and is therefore less prone to misinterpretation. Design (c), where the 2D material is in direct ohmic contact with the transmission line, offers the most reliable conditions for observing a true Drude response, assuming a perfect ohmic contact can be made to the sample, which is generally difficult to achieve (see the SI). However, for certain sample widths or conductivities, plasmonic resonances can emerge on top of the Drude response from the *W*_2_ region. Device dimensions should therefore be chosen to prevent the plasmons from obscuring or inhomogeneously broadening the Drude response (see the SI).

Having established how sample size and Drude weight govern the appearance of a Phantom-Drude response in design (a), we now examine the role of linewidth. In principle, self-cavity modes with infinitely narrow linewidths would produce clearly distinguishable resonances, eliminating confusion with a Drude response (see the SI). However, as the linewidth increases, whether due to enhanced intrinsic electron scattering or inhomogeneous broadening, the ability to resolve individual modes diminishes, making a broad spectrum increasingly difficult to interpret. In the following, we examine two key mechanisms responsible for inhomogeneous linewidth broadening in on-chip THz spectroscopy.

## Mechanism 2: Inhomogeneous Linewidth Broadening through Irregularly Shaped 2D Materials

Since plasmonic oscillations of the current density extend from one sample edge to the other, the resonance frequencies of the self-cavity modes are determined not only by the transmission line geometry but also by the overall sample width (the length is largely irrelevant; see ref 22). If the sample extends irregularly beyond the transmission line, with nonrectangular boundary conditions, different regions along the THz pulse propagation direction will support self-cavity modes with varying wavelengths and resonance frequencies. As illustrated in Figure 3, such a nonuniform 2D material can be conceptually divided into slices, each exhibiting a distinct plasmonic wavelength and thus resonance frequency. The superposition of these detuned modes leads to an inhomogeneous broadening. When the overall resonance frequencies are sufficiently low, this can further give rise to a Phantom-Drude response, as evidenced by the measured data in Figure 3d,e.

**Figure 3:**
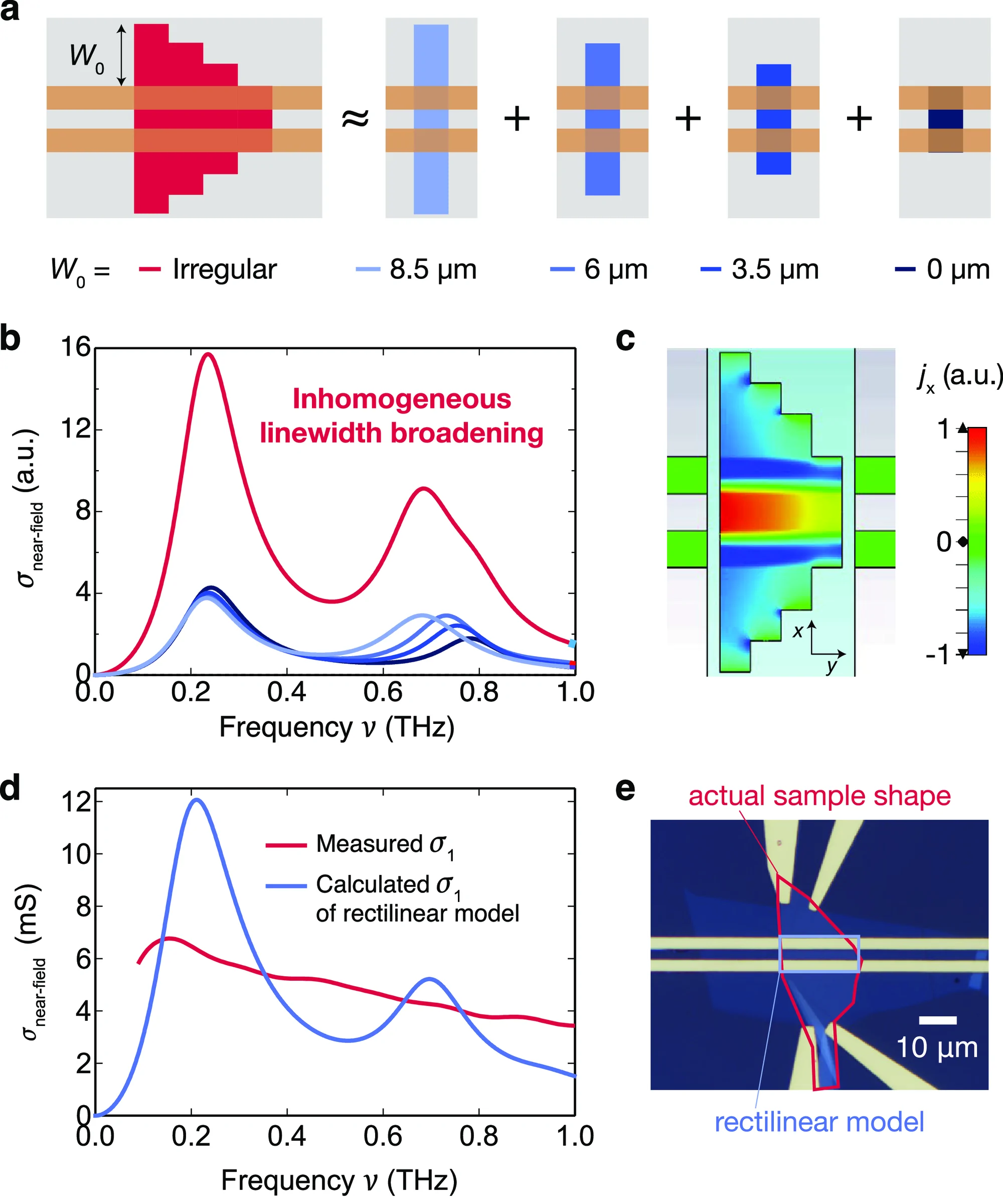
Inhomogeneous linewidth broadening due to irregularly shaped 2D materials: (a) An irregularly shaped 2D material embedded in on-chip THz spectroscopy can be decomposed into slices of fixed width (here, 2.5 μm). Since the frequencies of the self-cavity modes strongly depend on the local width *W*_0_ of the part of the 2D material extending the transmission line, the overall near-field optical conductivity spectrum of the irregular flake can be approximated as the sum of the responses from each individual slice. (b) Full 3D electromagnetic simulations are performed for both the entire irregular flake and its constituent slices. (c) Full 3D electromagnetic simulations of the current density distribution in the irregularly shaped sample at 0.686 THz and a phase of 103°. Distinct plasmonic modes of different wavelengths, and thus different resonance frequencies, form in different slices of the sample, whose combined response gives rise to the inhomogeneously broadened high-frequency peak shown in (b). Sample parameters: *W*_1_ = *W*_2_ = 3 μm, *D* = 24.5 mS × THz × rad, *d*_hBN_ = 25 nm, and γ = 1.5/(2π) THz. (d) Measured near-field conductivity of an irregularly shaped graphite sample (red), shown in (e), compared with the calculated near-field conductivity assuming an ideal rectangular geometry (rectilinear model). Sample parameters: *W*_1_ = 2.9 μm, *W*_2_ = 3.0 μm, *d*_gr_ = 5.5 nm, and *d*_hBN_ = 16.5 nm.

This issue can be mitigated by using precisely shaped, rectangular 2D materials, achievable through anodic oxidation lithography^51^ or etching methods.

## Mechanism 3: Inhomogeneous Linewidth Broadening through Hybridization with Gate Plasmons

To fully explore the rich physics of vdW materials, electrostatic gates are essential for enabling *in situ* tuning of the carrier density. However, incorporating such gates into on-chip THz spectroscopy introduces additional complexity. Since electrostatic gating requires the gate material to be at least weakly conductive to facilitate the formation of mirror charges and modulate the carrier density, it unavoidably introduces THz absorption. To mitigate this effect, different groups have adopted a variety of strategies. Some have employed low-conductive gates, which exhibit a weak response in the THz regime.^24,26,52,53^ Others have implemented photogating techniques, in which a semiconducting transition-metal dichalcogenide (TMD) gate becomes conductive only under optical excitation.^25^ Alternatively, metallic graphite gates have been integrated into on-chip THz circuitry with a dedicated reference arm (Figure 1a),^22,31^ allowing direct characterization of the metallic gate response when the vdW material is at charge neutrality or in an insulating state.

A common strategy to mitigate gate contributions to the THz signal is to subtract them. However, as we demonstrate below, this approach can introduce complications. Plasmons can form in both vdW material and gate that hybridize strongly to form symmetric (in-phase) and antisymmetric (180° out-of-phase) plasmonic modes (see Figure 4a), that, depending on the conductivities and separations of the two layers, can significantly differ in resonance frequencies and spectral weight from their uncoupled counterparts (see Figure 4b). In particular, when a plasmon can form in the gate and it has a low Drude weight (*D*_gate_/*D*_vdW material_ ≪ 1), an avoided crossing appears where the uncoupled gate and vdW material modes would otherwise cross in frequency. This avoided crossing signifies strong hybridization, highlighting that low-conductive gates can complicate the extraction of intrinsic vdW material properties. For example, a low-conductive gate’s plasmonic resonance cannot be removed from a measured conductivity spectrum by simple subtraction, as such a procedure can even produce unphysical negative effective conductivities.

**Figure 4:**
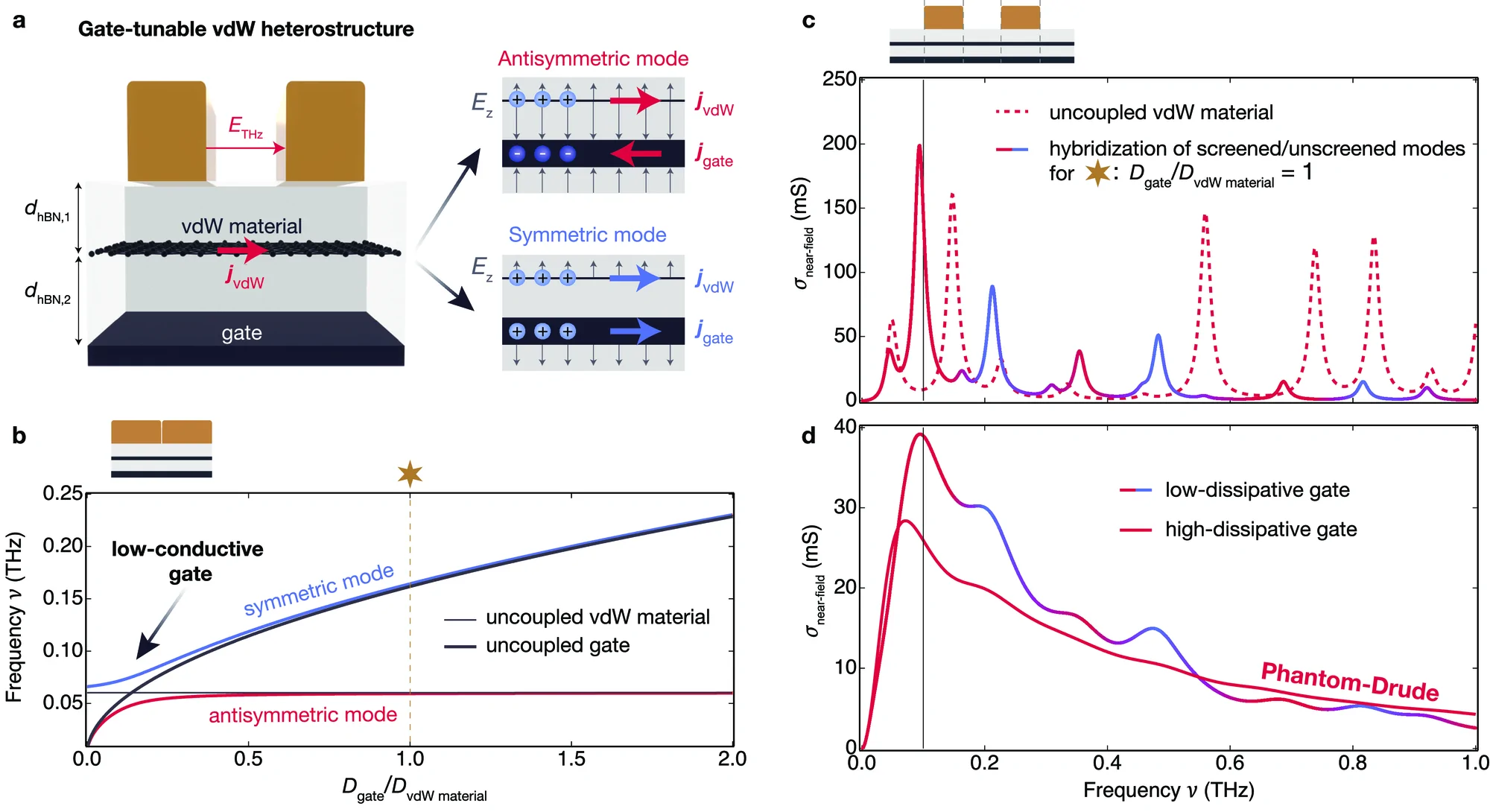
The challenge of low-conductive gates: (a) In on-chip THz spectroscopy of gate-tunable vdW heterostructures, plasmonic modes in the vdW layer and nearby gate strongly couple due to their nanometer separation, forming hybrid symmetric (in-phase) and antisymmetric (out-of-phase) excitations. (b) Calculated resonance frequencies of hybrid and uncoupled modes versus gate-to-vdW material Drude weight ratio of a perfectly screened heterostructure (transmission line design (b) (see Figure 2b)). Highly conductive gates yield weak hybridization, while low-conductive gates cause strong mode mixing and large frequency shifts. (c) Near-field conductivity spectra for gate-tunable vdW heterostructures in a transmission line of design (a) (see Figure 2a) calculated for an unrealistically low scattering rate to highlight the individual resonances. Two cases are compared: a perfectly screened vdW layer with a thick metallic gate (dashed red) and a vdW layer with a lower conductive gate where *D*_gate_/*D*_vdW material_ = 1 (red-blue). In the latter, antisymmetric (vdW material-like, red) and symmetric (gate-like, blue) hybrid plasmon modes appear. The antisymmetric modes shift to lower frequencies due to coupling with the symmetric modes, a result of resonant interaction at the dielectric boundaries provided by the metal trace edges, as described in ref 22 (d) When the scattering rate is increased to an experimentally realistic value of γ_gate_ = γ_vdW material_ = 0.12 THz, distinct spectral features from multiple resonances remain visible (red-blue curve). However, if the gate’s scattering rate becomes much larger than that of the vdW material, either due to intrinsic higher scattering or inhomogeneous linewidth broadening, both the symmetric (blue) and antisymmetric (red) modes broaden significantly, merging into a smooth, featureless Phantom-Drude response (red curve). Sample parameters for panel (b): momentum *q* = π/(39 μm), *D*_vdWmaterial_ = 43.35 mS × THz × rad, *d*_hBN,1_ = 20 nm, and *d*_hBN,2_ = 100 nm. Sample parameters for panels (c) and (d): *W*_0_ = *W*_1_ = *W*_2_ = 13 μm, *d*_hBN,1_ = 20 nm, *d*_hBN,2_ = 100 nm, and *D*_vdWmaterial_ = *D*_gate_ = 34.05 mS × THz × rad. Scattering rates in (c): γ_gate_ = γ_vdWmaterial_ = 0.02 THz. Scattering rates in (d): γ_gate_ = γ_vdWmaterial_ = 0.12 THz (red-blue) and γ_gate_ = 5 × γ_vdWmaterial_ = 0.60 THz (red).

In contrast, when the layers are well separated by a thick hBN layer and/or the gate is much more conductive than the 2D material, the modes behave approximately as independent gate and 2D material plasmons (see Figure 4b and also ref 22).

However, symmetric and antisymmetric modes only behave as uncoupled gate and 2D material plasmons if the gate-tunable heterostructure is placed in a homogeneously screened environment, such as in transmission line design (Figure 2b). When the system follows transmission line design (a) (Figure 2a), the situation becomes more complex. Symmetric and antisymmetric modes can form both in the screened regions beneath the metal traces and also in the unscreened regions in the transmission line gap and outside the transmission line, with different dispersion relations (see the SI). As the antisymmetric unscreened modes in the *W*_0_ and *W*_2_ regions (see Figure 4a) have comparable resonance frequencies to the screened antisymmetric and symmetric modes in the *W*_1_ regions, they can resonantly enhance the coupling of the screened modes, even of different momenta.^22^ In some cases, this can push the system into the *ultrastrong coupling* regime,^54,55^ where the hybrid mode splitting exceeds 10% of the original resonance frequencies.

This type of coupling has a key consequence: in a homogeneously screened heterostructure, symmetric and antisymmetric plasmonic modes can closely resemble the uncoupled modes of the gate and the vdW material. As shown in Figure 4b, when the gate and vdW material have comparable Drude weights (*D*_gate_/*D*_vdW material_ ≈ 1), the resonance frequency of the antisymmetric (vdW material-like) mode remains essentially unchanged, even as the gate conductivity increases beyond this ratio. In contrast, this behavior changes dramatically in a micropatterned dielectric environment, such as that introduced by transmission line design (a). As illustrated in Figure 4c, the antisymmetric mode, originally resembling that of the uncoupled vdW material (dashed red), is shifted to lower frequencies (solid red) due to hybridization with symmetric, gate-like modes (solid blue), even at *D*_gate_/*D*_vdW material_ = 1.

Furthermore, hybridization with the gate affects not only the resonance frequencies but also the linewidths of the vdW material-like modes. If a plasmon hosting gate is chosen to exhibit an intentionally high scattering rate of several THz such that the gate conductivity alone would exhibit a relatively flat and featureless response at frequencies below 1 THz, then this scattering rate will broaden the antisymmetric mode associated with the 2D material, leading to an artificially broadened spectral response of the vdW material. When many such broadened hybrid modes lie within the measurement bandwidth, their superposition can again lead to a Phantom-Drude response (Figure 4d). Fitting such a broadened spectrum with a Drude model yields apparent parameters γ_Phantom_ = 0.42 THz and *D*_Phantom_ = 63.44 mS × THz × rad that deviate by over 400% from the true intrinsic values of γ = 0.1 THz and *D* = 43.35 mS × THz × rad (see the SI).

These findings highlight that while low-conductive gates may initially seem like a straightforward choice for on-chip THz spectroscopy, they can complicate data interpretation by strongly modifying the response of a vdW material and potentially giving rise to a Phantom-Drude response. Such gates are most effective when intentionally used for cavity control protocols, provided they are carefully engineered in thickness and dimensions to limit the number of hybridizing self-cavity modes within the experimental sensitivity range.^22^

In contrast, to probe the intrinsic response of a gate-tunable vdW material with minimal disturbance, a highly conductive gate, such as a 20 to 30 nm thick graphite flake, separated from the 2D material by a ∼100 nm hBN spacer and embedded in a transmission line of design (b), provides an effective solution. In this “sensing cavity” configuration,^22^ coupling and linewidth broadening are strongly suppressed, and the gate contribution can be reliably subtracted using the on-chip THz architecture with built-in referencing (Figure 1a).^31^ Graphite flakes are widely employed in high-quality DC transport experiments for their reliability and low disorder (Figure S11 of ref 10) and allow symmetric, precise carrier density control for both electron and hole doping down to millikelvin temperatures. This is advantageous, as it indicates that the most effective approach for on-chip THz spectroscopy is to use standard graphite gates for vdW heterostructures, while carefully considering boundary conditions and flake thicknesses.

## Discussion and Outlook

By demonstrating that Phantom-Drude responses, which arise due to the superposition of plasmonic modes of different momenta, can emerge in on-chip THz near-field spectra due to choices in sample size, transmission line geometry, vdW material, gate shapes, and gate material, this article highlights an important response regime. Crucially, plasmonic cavity responses should not be regarded as experimental artifacts to be avoided, but rather as the physical electrodynamic response of the system under the finite-momentum fields imposed by the device geometry. In on-chip THz spectroscopy, the measured quantity is the near-field conductivity, which relates the current density to the local electric field via *j* = σ*E*. The observed resonances therefore directly reflect how currents flow in the material in the presence of electromagnetic boundary conditions and geometric confinement. In this sense, plasmonic self-cavity modes are not a distortion of the response, but its natural manifestation in a finite-momentum electrodynamic environment. When engineered outside the Phantom-Drude regime, they provide direct access to finite-momentum electrodynamics and constitute a powerful probe of emergent phenomena in 2D materials.^22,23,30,46^

We therefore provide design guidelines that enable selective access to either genuine Drude behavior or cavity electrodynamics in gate-tunable vdW heterostructures. Importantly, in the absence of uncontrolled gate-induced hybridization, the analytical theory and numerical methods developed here establish a direct mapping between a measured near-field response and the intrinsic 2D conductivity of a material. Under these conditions, the framework enables the unambiguous distinction between genuine Drude behavior and Phantom-Drude responses arising from plasmonic cavity effects, making it an indispensable tool for the interpretation of THz near-field spectra. Although this article focused on responses of metallic 2D materials, the same principles extend to systems supporting other collective modes such as phonons, excitons, or plasmons in strange metals and superconductors.^30^

Looking ahead, the analytical framework developed for 2D materials in on-chip THz spectroscopy^30^ opens new avenues for tailoring sample designs to probe intricate spectral signatures of emergent phenomena in 2D quantum materials. Importantly, while our discussion centers on a specific on-chip THz spectroscopy circuit featuring two parallel metal traces, the underlying design principles for vdW heterostructures are broadly applicable. These principles can be readily adapted to other on-chip THz circuit architectures and may provide valuable guidance for a wide range of near-field techniques.

## Supplementary Material



## Data Availability

The data that support the findings of this study are available from the corresponding authors upon request.

## Notes

Additional details of the analytical theory, experimental validation of the analytical theory, data fitting of calculated Phantom-Drude response in Figure 4d, experimental demonstration of a Phantom-Drude response, parameter sensitivity, details of full 3D electromagnetic simulations, and additional full 3D electromagnetic simulations (PDF)
