# Supplementary material for: Response Regimes in On-Chip THz Spectroscopy
Source: ACS Photonics. 2026 Jul 3;13(16):4530–8. doi: 10.1021/acsphotonics.6c00409 (PMC13495959; doi:10.1021/acsphotonics.6c00409)
Supplement: Supplementary file 1 [file ph6c00409_si_001.pdf]

SUPPORTING INFORMATION

Response regimes in on-chip THz spectroscopy

Gunda Kipp,[1,2,*] Marios H. Michael,[3,1,2] Alexander M. Potts,[1,2,4] Dorothee Herrmann,[1,2] Toru Matsuyama,[1,2] Guido Meier,[1,2] Matthew W. Day,[1,2,4] Hope M. Bretscher,[1,2,4] and James W. McIver[1,2,4,†]

[1] *Max Planck Institute for the Structure and Dynamics of Matter, Luruper Chaussee 149, 22761 Hamburg, Germany*
[2] *Center for Free-Electron Laser Science (CFEL), Luruper Chaussee 149, 22761 Hamburg, Germany*
[3] *Max Planck Institute for the Physics of Complex Systems, Nöthnitzer Straße 38, 01187 Dresden, Germany*
[4] *Department of Physics, Columbia University, 538 West 120th Street, New York, NY 10027, USA*
(Dated: June 19, 2026)

1. ANALYTICAL THEORY

1.1. General principle

Here, we summarize the key ideas of the analytical theory used in the main text. A detailed discussion may be found in Refs.[1,2].

The theory solves Maxwell's equations to describe the electrodynamics of 2D materials embedded in on-chip THz spectroscopy with a cross-section as shown in Fig. 1, where the metallic transmission lines are modeled as perfect electric conductors. A symmetric sample can be divided into five regions, where W_0 is the width of the two unscreened 2D material regions extending beyond the transmission line, W_1 is the width of each metal trace and W_2 is the width of the transmission line gap. Plasmons are driven by the THz pulse's electric field throughout all regions of the 2D material, with different dispersion relations for screened and unscreened plasmons (see main text). By solving Maxwell's equations for the transmitted ($t_{0,1,2}$) and reflected ($r_{0,1,2}$) waves at the dielectric boundaries indicated by the grey dashed lines in Fig. 1, and assuming continuity of the current density ($j_{0,1,2}$) and electrostatic potential across these boundaries, as well as vanishing current density ($j_0 = 0$) at the sample edges, one can calculate both the effective plasmonic current-density profile in the integrated 2D material and its corresponding near-field optical conductivity.

Depending on sample size, transmission line design and the conductivity of the sample, the effective near-field optical conductivity approaches a limit, where it resembles a Drude response, or it is characterized by multiple finite-momentum plasmonic resonances, as quantified by the feedback factor F. When $F \approx 0$, a Drude response is observed.

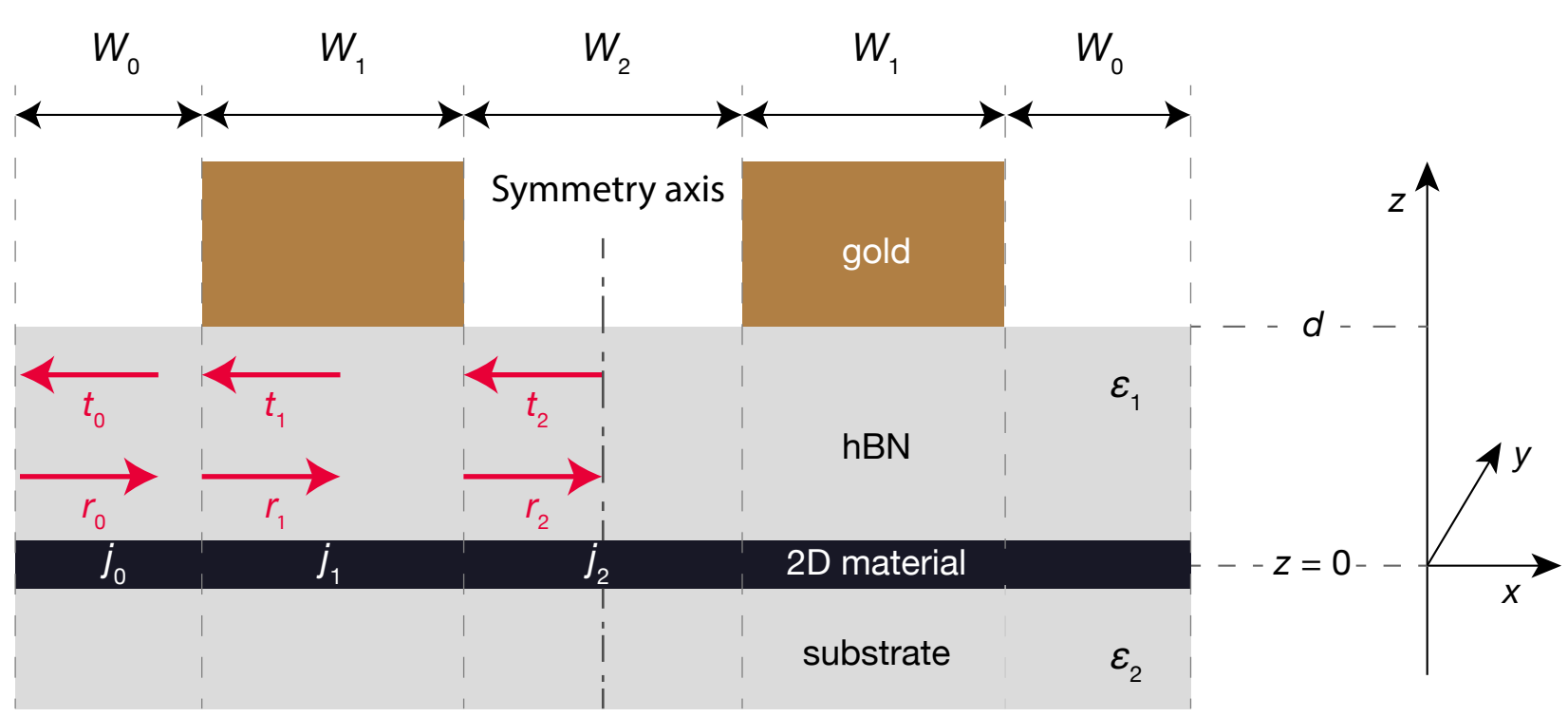

FIG. 1: **Cross-section of an on-chip THz device:** A 2D material encapsulated with an insulating layer such as hBN is placed on an insulating substrate. The metal traces that form the transmission lines are deposited on top. The analytical theory describes the effective near-field conductivity and current density profiles by solving Maxwell's equations for transmitted $t_{0,1,2}$ and reflected $r_{0,1,2}$ waves at the dielectric boundaries provided by the sample's edges and metal trace edges (grey dashed lines).

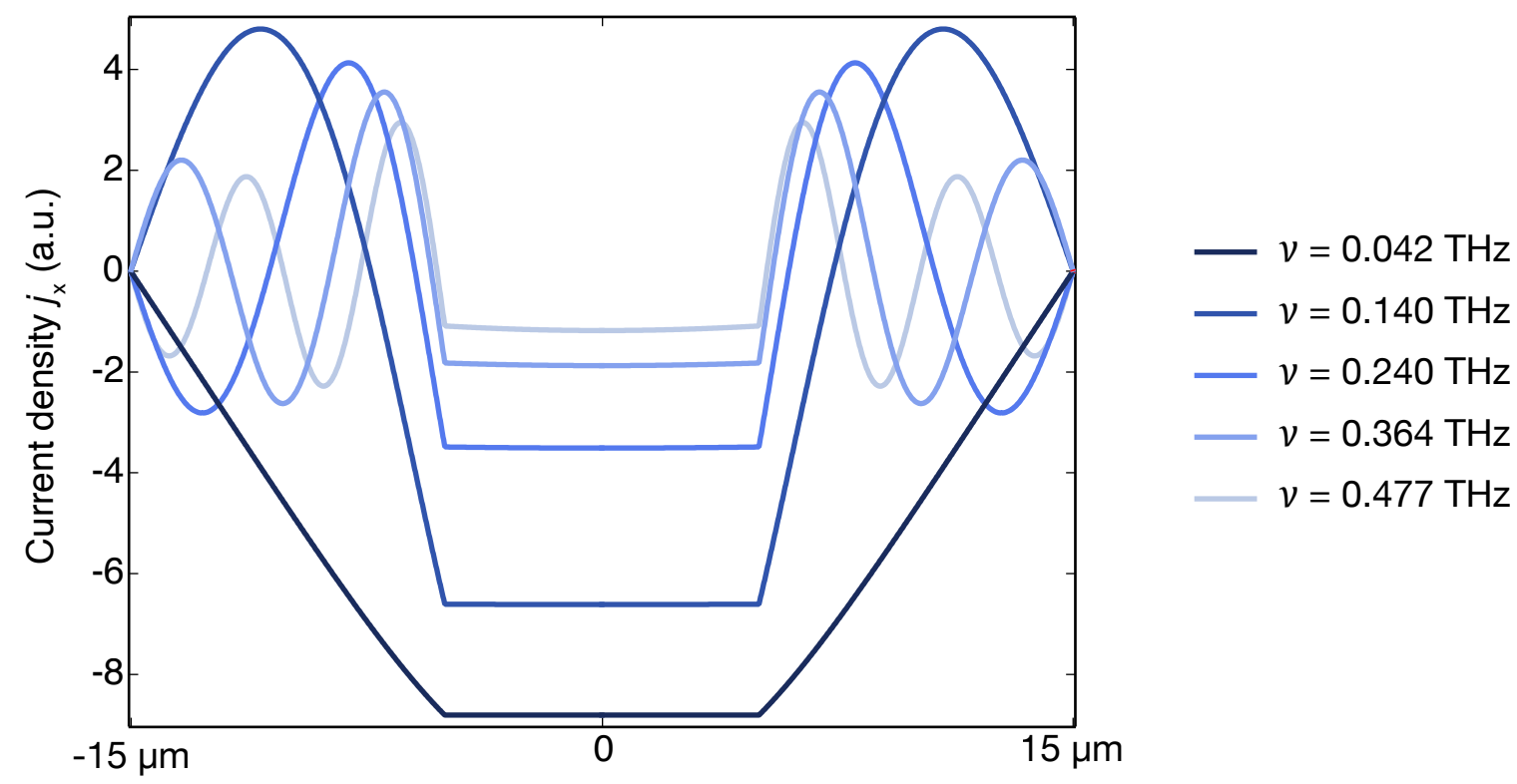

FIG. 2: **Current density profiles of the plasmonic resonances shown in Fig. 1b:** The current density profiles are calculated with the analytical theory for the five lowest-frequency resonances.

When $F \neq 0$, the system exhibits a plasmonic cavity or Phantom-Drude response. The expression for F is given in the main text for $W_0 = 0$. For $W_0 \neq 0$, it is given by:

$$F = \frac{\mathrm{sinc}\,(q_{\mathrm{un}} W_2/2)}{-\cos(\frac{q_{\mathrm{un}} W_2}{2}) + \frac{q_{\mathrm{sc}}(q_{\mathrm{un}}\cos(q_{\mathrm{sc}}W_1)\sin(q_{\mathrm{un}}W_0) + q_{\mathrm{sc}}\cos(q_{\mathrm{un}}W_0)\sin(q_{\mathrm{sc}}W_1))\sin(q_{\mathrm{un}}W_2/2)}{q_{\mathrm{un}}(q_{\mathrm{sc}}\cos(q_{\mathrm{un}}W_0)\cos(q_{\mathrm{sc}}W_1) - q_{\mathrm{un}}\sin(q_{\mathrm{un}}W_0)\sin(q_{\mathrm{sc}}W_1))}}. \tag{1}$$

1.2. Current density profiles of inset of Fig. 1b

In Fig. 2, the analytical theory is used to calculate the current density profiles of the plasmonic resonances underlying Fig. 1 of the main text, whose superposition gives rise to the Phantom-Drude response.

1.3. Symmetric and antisymmetric modes in gated heterostructures

In case of a heterostructure comprising a 2D material and an electrostatic gate, plasmons are excited in both the 2D material and the gate. Due to the close proximity of the two layers, the plasmons hybridize strongly to form symmetric (in-phase) and antisymmetric (out-of-phase) modes. As a consequence, symmetric and antisymmetric modes form in each region, with different dispersion relations for screened and unscreened modes. For a detailed derivation of the analytical framework describing this dual-layer structure, see Refs.[1,2].

The calculations in Fig. 4b of the main text were performed using the following formulas:

$$\nu_{sc,sym} = \sqrt{\frac{2q^2 d_1 d_2 D_T D_B}{(2\pi)^2\left((d_2\epsilon_0\epsilon_1 D_B + d_1\epsilon_0\epsilon_1(D_T + D_B)) + \sqrt{(4d_1 d_2 \epsilon_0^2\epsilon_1^2 D_T D_B - (d_2\epsilon_0\epsilon_1 D_B + d_1\epsilon_0\epsilon_1(D_T + D_B))^2)}\right)}}, \tag{2}$$

$$\nu_{sc,antisym} = \sqrt{\frac{2q^2 d_1 d_2 D_T D_B}{(2\pi)^2\left((d_2\epsilon_0\epsilon_1 D_B + d_1\epsilon_0\epsilon_1(D_T + D_B)) - \sqrt{(4d_1 d_2 \epsilon_0^2\epsilon_1^2 D_T D_B - (d_2\epsilon_0\epsilon_1 D_B + d_1\epsilon_0\epsilon_1(D_T + D_B))^2)}\right)}}, \tag{3}$$

$$\nu_{vdW} = \sqrt{\frac{q^2 D_T}{(2\pi)^2 \epsilon_0 \epsilon_1 \left(\frac{1}{d_1} + \frac{1}{d_2}\right)}}, \tag{4}$$

$$\nu_{gate} = \sqrt{\frac{q^2 D_B (d_1 + d_2)}{(2\pi)^2 \epsilon_0 \epsilon_1}}, \tag{5}$$

where $\nu_{sc,sym}$ is the resonance frequency of the screened symmetric mode, $\nu_{sc,antisym}$ is the resonance frequency of the screened antisymmetric mode, ν_{vdW} is the resonance frequency of the uncoupled vdW material plasmon, screened from the metal traces above and from the gate below, ν_{gate} is the resonance frequency of the uncoupled gate plasmon, screened from the metal traces above, with the momentum $q = 2\pi/\lambda$, the top hBN thickness d_1, the bottom hBN thickness d_2, the Drude weight of the top layer (vdW material) $D_T = D_{\text{vdW material}}$, the Drude weight of the bottom layer (gate) $D_B = D_{\text{gate}}$, the dielectric constant of hBN ϵ_1 and the vacuum permittivity ϵ_0. The following values were used for the calculation in Fig. 4b of the main text: $q = \pi/(39\,\mu\text{m})$, $d_1 = 20\,\text{nm}$, $d_2 = 100\,\text{nm}$, $D_T = 43.35\,\text{mS} \times \text{THz} \times \text{rad}$, $\epsilon_1 = 3.7$.

The calculations in Fig. 4c,d of the main text were performed using the full analytical theory of dual-layer structures discussed in Refs.[1,2].

1.4. Ohmic contact within the analytical theory framework

The ohmic contact scenario can be captured by allowing charge flow between the 2D material and the metal traces in the W_1 regions. This is achieved by imbuing the layer separating the 2D material and gold described by the permittivity ϵ_1 with a conductive response. For extremely thin hBN layers for example, the conductive response can represent tunneling between the material and the metal along the z-direction. In the W_1 regions, the permittivity ϵ_1 is then replaced by:

$$\epsilon_{1,\text{ohmic}} = \epsilon_1 + \frac{\sigma_{\text{DC,z}}}{-i\omega}. \tag{6}$$

At very small frequencies or equivalently very large $\sigma_{\text{DC,z}}$, such that $\frac{\sigma_{\text{DC,z}}}{-i\omega} \to \infty$, we reach the conductivity limit, where q_{sc} obtains a large imaginary part, causing plasma waves traveling in the screened region to quickly decay due to the ohmic contact. Mathematically, this causes $\cos(q_2 W_2/2) \propto e^{-\text{Im}\{q_2\} W_2/2} \to 0$. In this case, the feedback factor, for $W_2 = 0$, $|q_{un}| \ll |q_{sc}|, 2/W_2$ is found to be:

$$F_{\text{ohmic}} \overset{\sigma_{\text{DC,z}} \to \infty}{\approx} \frac{2}{W_2 q_1} \propto \frac{1}{\sqrt{\sigma_{\text{DC,z}}}}, \tag{7}$$

which vanishes as the inverse $\sqrt{\sigma_{\text{DC,z}}}$. In the perfect ohmic contact case, one can imagine that the 2D material is not separated from the metal traces and is instead directly contacted to the gold. The large conductivity of the gold suppresses F and leads to $\sigma_{\text{near-field}} \approx \sigma_{\text{Drude}}$.

We note that achieving high-quality ohmic contact is generally challenging, both for vdW materials and even for sputtered films. For example, in Ref.[3], a superconducting thin film integrated into on-chip THz circuitry in direct contact with the transmission line developed a thin, leaky oxide layer at the interface. As a result, the measured response comprised a superposition of Cooper plasmonic modes and a conventional DC superconducting contribution.

For vdW materials, the situation is further complicated by the requirement of a gate that is electrically isolated from the conducting layer and, consequently, also from the transmission line. Thus, even if ohmic contact between the vdW material and the transmission line were successfully established and a Drude response could be measured, the gate would still support plasmonic self-cavity resonances that must be accounted for in the overall signal of the heterostructure.

In practice, this suggests that, in the absence of ideal ohmic contact, it is often more robust to deliberately target and exploit the plasmonic cavity response rather than attempting to isolate a purely Drude-like regime.

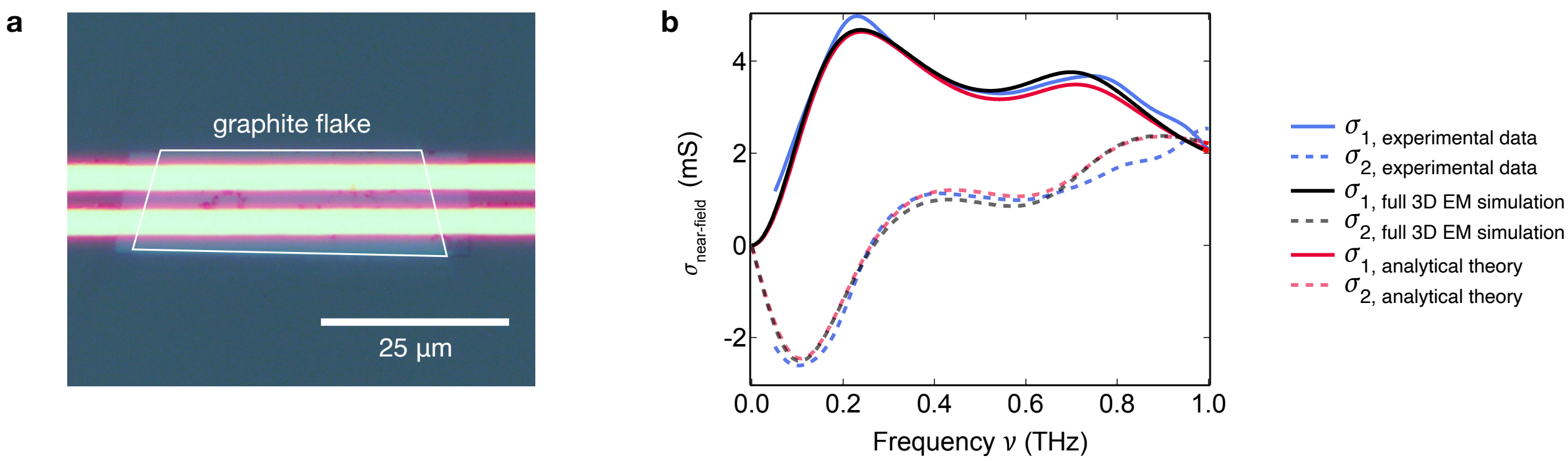

FIG. 3: **Experimental validation of the analytical theory: a** Optical micrograph of a graphite-hBN heterostructure embedded into on-chip THz circuitry. Sample dimensions: $l = 38\,\mu\mathrm{m}$, $W_0 = 1\,\mu\mathrm{m}$, $W_1 = 3.33\,\mu\mathrm{m}$, $W_2 = 2.56\,\mu\mathrm{m}$, $d_{\mathrm{gr}} = 5\,\mathrm{nm}$ and $d_{\mathrm{hBN}} = 24\,\mathrm{nm}$. **b** Experimentally-measured real and imaginary parts of the near-field optical conductivity of the sample shown in (a) as function of frequency in comparison to calculations of the analytical theory and full 3D electromagnetic simulations. Figure and data adapted from[2] under a CC BY 4.0 license.

1.5 Validation of the analytical theory

To validate the analytical theory, Figure 3 demonstrates strong agreement among experimental data, full 3D electromagnetic simulations, and calculations performed with the analytical theory.

1.6. Simulation of an irregularly shaped sample within the analytical theory framework

While the irregularly shaped sample discussed in Fig. 3 of the main text was analyzed through full 3D electromagnetic simulations, the analytical theory can also be used to approximate the effective conductivity of an irregularly shaped sample:

$$\sigma_{\text{irregularly shaped sample}} = \int \sigma_{\text{near-field}}(\nu, W_0) P(W_0) dW_0, \tag{8}$$

where $\sigma(\nu, W_0)$ is the frequency- and W_0-dependent near-field conductivity and $P(W_0)$ is the distribution of the width W_0 across the device.

1.7. Drude fit of the Phantom-Drude response in Fig. 4d

The Drude fit of the calculated Phantom-Drude reponse shown in Fig. 4d that emerges through hybridization with a dissipative gate in a transmission line design with $W_1 \approx W_2$ is shown in Fig. 4. The Drude fit parameters differ from the intrinsic vdW material parameters by up to $\sim$400 %.

1.8. Experimental demonstration of a Phantom-Drude response

An experimental demonstration of a Phantom–Drude response, measured on a wide and thin graphite flake, is presented in Figure 5. Fitting the measured data (blue) with a Drude model (red) yields parameters such as a scattering rate of 0.31 THz and a DC conductivity of 17.1 mS. However, comparison with calculations based on the analytical near-field theory (black) and the intrinsic conductivity of the sample (gold) reveals a pronounced discrepancy: the Drude fit to the Phantom–Drude response (red) deviates significantly from the intrinsic Drude

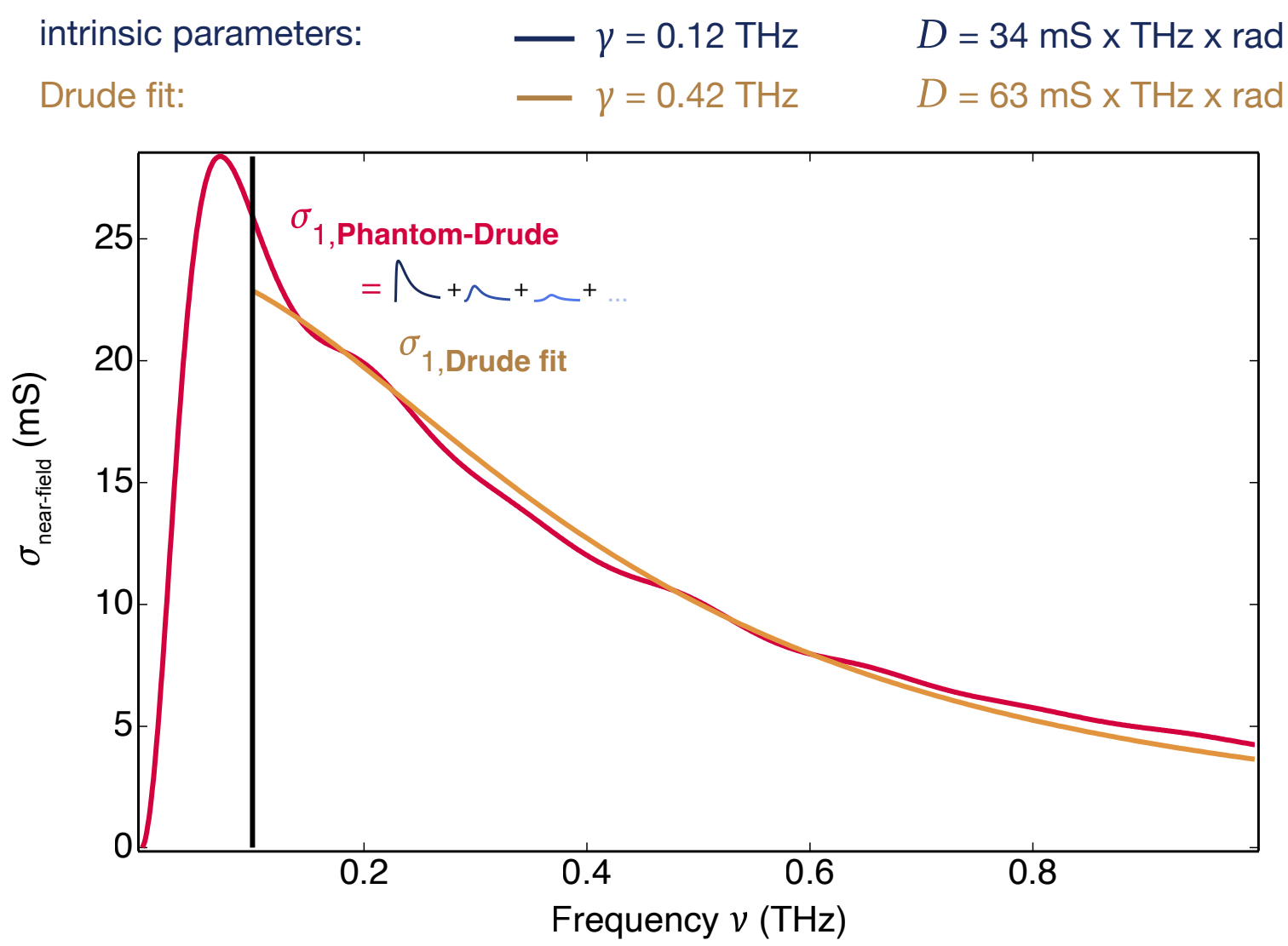

FIG. 4: **Drude fit of the Phantom-Drude response in Fig. 4d:** The Phantom-Drude reponse of gate-hybridization origin is fit to a Drude model in the frequency range of 0.1 THz to 1 THz.

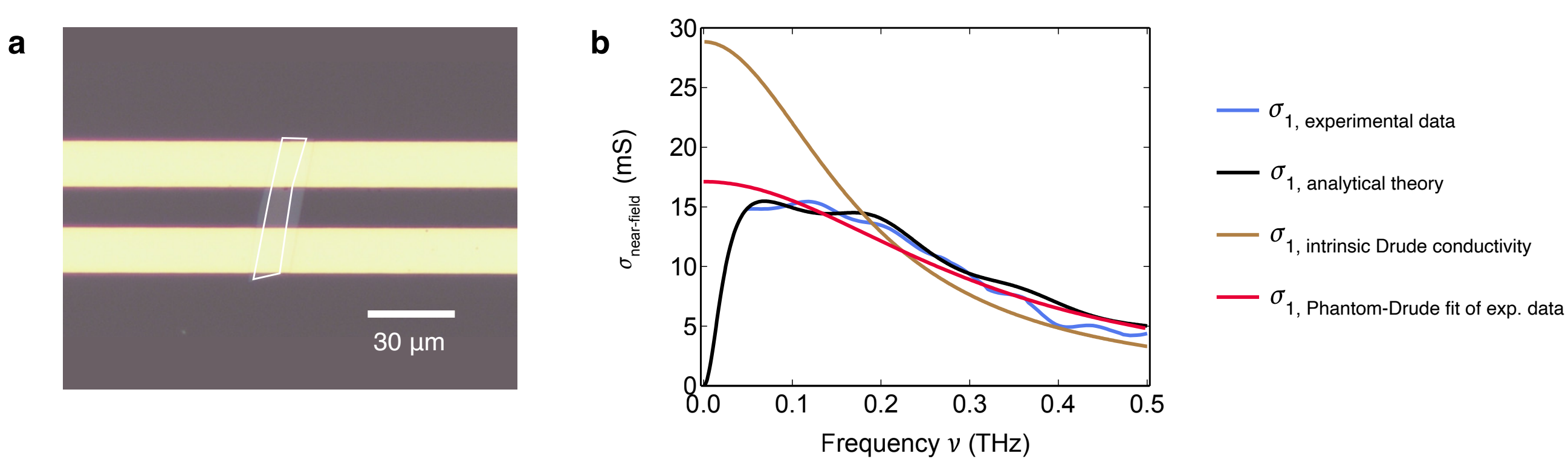

FIG. 5: **Experimental demonstration of a Phantom-Drude response: a** Optical micrograph of a graphite-hBN heterostructure embedded into on-chip THz circuitry. Sample dimensions: $W_1 = 16\,\mu\text{m}$, $W_2 = 14\,\mu\text{m}$, $d_{\text{graphite}} = 7\,\text{nm}$ and $d_{\text{hBN}} = 32\,\text{nm}$. **b** Experimentally measured (blue) and calculated (black) near-field optical conductivities of the sample in (a) as function of frequency. A Drude-fit of the experimental data (red) differs significantly from the intrinsic Drude-conductivity of the graphite sample (gold). Figure and data partially adapted from[1] under a CC BY 4.0 license.

conductivity (gold), for which the scattering rate and DC conductivity are 0.18 THz and 28.8 mS, respectively.

1.9. Sensitivity of plasmonic self-cavity resonance frequencies to variations in sample parameters

The near-field conductivity spectrum of a sample is highly sensitive not only to its intrinsic conductivity, but even more so to the parameters of its dielectric environment. For this reason, all parameters used for measured, calculated, and simulated samples are specified with high precision throughout the manuscript. To determine which regime applies to a specific sample, the effective near-field conductivity has to be evaluated using Eqs. (1) and (2) of the main text.

To further build intuition for the influence of individual parameters, we illustrate in Figure 6 how the resonance frequency, specifically of the lowest-frequency (“m = 0”) plasmonic mode, shifts when a single parameter of the

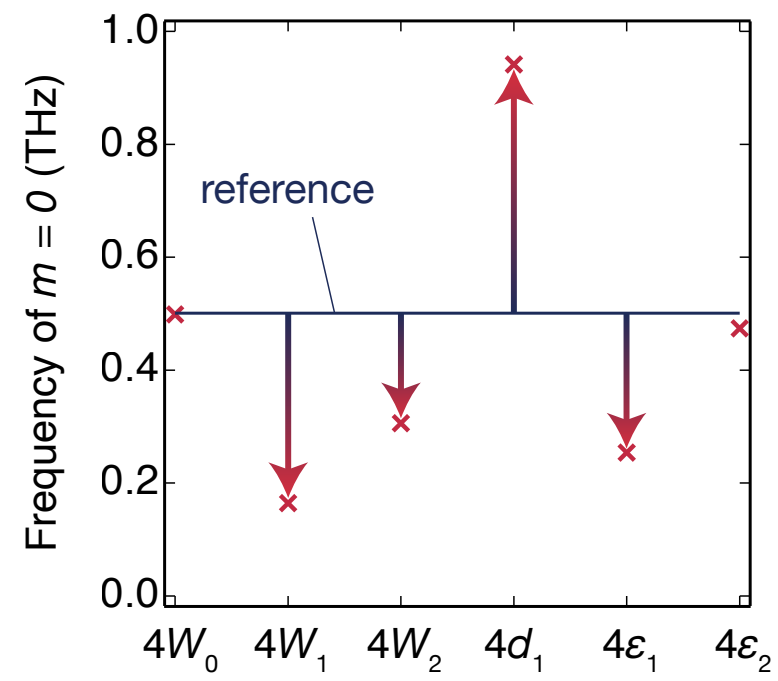

FIG. 6: **Variation of the resonance frequency of the lowest frequency "m=0") mode towards parameter changes of the dielectric environment:** When the parameters of a sample with $W_0 = 3\,\mu$m, $W_1 = 3\,\mu$m and $W_2 = 4\,\mu$m, $d_{\mathrm{gr}} = 15.33\,$nm, $d_1 = d_{\mathrm{hBN}} = 35\,$nm are increased by a factor of four, the original lowest-frequency resonance of the sample, with frequency $\nu_0 = 0.5\,$THz, is changed as indicated by the arrows.

dielectric environment is varied by a factor of four.

Please note that the $m = 0$ mode exhibits only weak sensitivity to variations in W_0, as illustrated in Figure 6. In contrast, higher-order modes depend strongly on W_0, which can result in inhomogeneous linewidth broadening in samples with irregular geometries, as discussed in Fig. 3 of the main text.

2. FULL 3D ELECTROMAGNETIC SIMULATIONS

2.1. Method

The full 3D electromagnetic simulations are carried out with the frequency-domain solver in CST Microwave Studio[4], which employs the finite-element method (FEM) to solve Maxwell's equations. For more details about the method, see supplementary information of Refs.[1,2].

Importantly, the simulations account for the effective near-field conductivity of the integrated heterostructure and how efficiently a THz pulse interacts with the sample, thereby closely mimicking experimental conditions. This involves the consideration of a "filling factor", as discussed in the following section.

2.2. Filling factor

While a more detailed theoretical explanation of the filling factor is given in Refs.[1,2], we provide here a purely intuitive picture. The filling factor description is based on the fact that the detected near-field conductivity amplitudes from transmission data depends on how effectively the THz pulse interacts with a 2D material. The degree of interaction, governed by both the transmission line geometry and the thickness of the insulating layer between the transmission line and the 2D material, determines how much of the THz field is modified by the sample. A material placed in direct proximity to the transmission line significantly attenuates the transmitted THz pulse, whereas the same material separated by, for example, a thick hBN layer induces a markedly smaller transmission change. This filling factor thus emerges as a fundamental parameter: without accounting for it, neither Drude conductivities nor spectral weights of plasmonic resonances can be quantitatively inferred from experimental data.

We define the filling factor C_{filling} as (see Ref.[1])

$$C_{\mathrm{filling}} = \frac{\sigma_{\mathrm{sim}}}{l\sigma_{\text{near-field}}}, \tag{9}$$

with the simulated conductivity σ_{sim}, the sample length l in y-direction and the near-field conductivity (as calculated by the analytical theory) $\sigma_{\text{near-field}}$.

2.3. Simulations of ohmic contact design

As the analytical theory describes samples with an insulating layer between sample and transmission line, full 3D electromagnetic simulations were performed for a transmission line design in which the sample is placed in direct ohmic contact (see Fig. 2, main text). The simulations considered a sample with Drude weight $D = 6.28\,\mathrm{mS} \times \mathrm{THz} \times \mathrm{rad}$ and scattering rate $\gamma = 0.1\,\mathrm{THz}$, for various sample widths $W = 2W_1 + W_2$.

As shown in Fig. 7, a Drude response is observed for all sample widths. In addition, plasmonic resonances emerge on top of the Drude background for larger sample widths. These correspond to unscreened plasmons that form within the transmission line gap. As shown in Fig. 7b, the two lowest frequency resonances correspond approximately to $\lambda \approx \frac{2}{3}W_2$ and $\lambda \approx \frac{2}{5}W_2$ plasmons. The resonance frequencies of the unscreened plasmonic modes can therefore be approximated with:

$$\nu = \sqrt{\frac{D}{2\pi\lambda\epsilon_0(\epsilon_1 + \epsilon_2)}}. \tag{10}$$

with wavelengths $\lambda \approx 2W_2/a$, with $a = 3, 5, \ldots$. However, this approximation neglects the boundary conditions imposed by the ohmic contact to the transmission-line metal, resulting in deviations on the order of 100 GHz in the predicted resonance frequencies.

As the resonance frequencies of unscreened plasmons are much higher than those of screened or hybrid screened/unscreened modes, they enter the experimental bandwidth only for sufficiently large sample widths or for samples with low conductivity. This has important implications for sample design. Depending on the goal, one can choose the geometry for a given conductivity such that the plasmonic resonances lie outside the measurement bandwidth, ensuring a purely Drude response, or intentionally bring one plasmonic mode into the accessible range. In the latter case, the presence of a resolvable plasmonic resonance provides an additional tool for extracting intrinsic material parameters. This is particularly beneficial for samples with low scattering rates, where a significant fraction of the spectral weight lies below the measurement bandwidth and would otherwise be difficult to quantify. In contrast, if the sample parameters are not considered prior to fabrication, there is a risk of obtaining spectra with multiple overlapping plasmonic resonances in addition to the Drude response, resulting in a broad Phantom-Drude response that cannot be used to reliably extract intrinsic material properties.

2.4. Simulations of an irregularly shaped sample

To accurately capture the response of the irregularly shaped sample, full 3D electromagnetic simulations were performed for the structure shown in Fig. 2 of the main text, in addition to the individual slices of the structure. A sample length of $l = 10\,\mu\mathrm{m}$ (in x-direction) was chosen so that each section with a different total width has a length of $2.5\,\mu\mathrm{m}$. The current-density profiles of the x-directed current j_x at the two resonance-peak frequencies are shown for different phases in Figs. 8 and 9.

As shown in Fig. 8, the current density in the W_0 regions extending beyond the transmission line is essentially zero. Consequently, the resonance frequency of the lowest-momentum plasmonic mode is largely insensitive to the width W_0, and this mode does not experience significant inhomogeneous broadening due to the sample's irregular shape (see low-frequency mode in Fig. 3 of the main text).

In contrast, the higher-momentum modes exhibit a nonvanishing current density in the W_0 regions. As illustrated in Fig. 9, the plasmonic excitation spans the full sample width in the x-direction, with comparable current amplitudes in the W_0, W_1, and W_2 regions. As a result, these higher-momentum modes are strongly affected by the sample geometry, leading to pronounced inhomogeneous broadening in non-rectangular samples (see high-frequency mode in Fig. 3 of the main text).

2.5. Simulations of low-conductive samples

To validate the analytical theory and the conditions under which a phantom-Drude regime can emerge across a wide range of conductivities (or Drude weights), full 3D electromagnetic simulations were performed for a structure with $d_{\mathrm{hBN}} = 100\,\mathrm{nm}$, $W_0 = 0$ and $W_1 = W_2 = 10\,\mu\mathrm{m}$, while systematically varying D_{Drude}. As shown in Fig. 10, the analytical theory and full 3D electromagnetic simulations exhibit good agreement over a broad range of Drude

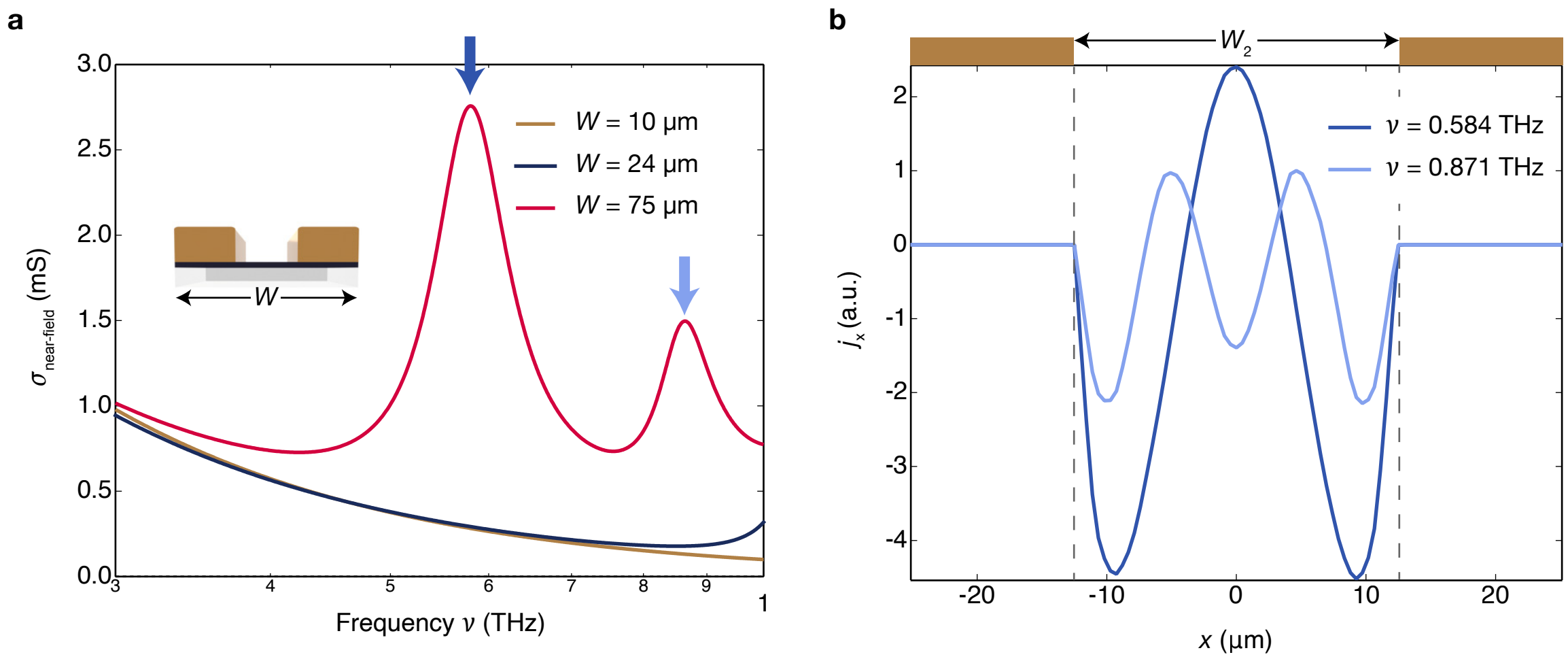

FIG. 7: **Current density profiles of the plasmonic resonances shown in Fig. 2d for the ohmic contact design: a** Full 3D electromagnetic simulations of the near-field optical conductivity as function of frequency for three different sample widths. Whereas Fig. 2d of the main text displays the response over the full 0.001 THz to 1 THz range, the plot shown here focuses on the resonances near the upper end of the bandwidth. Note that to extract $\sigma_{\text{near-field}}$ from the simulations, the following filling factors were considered: $C_{\text{filling}}(W = 10\,\mu\text{m}) = 326\,\text{mm}^{-1}$, $C_{\text{filling}}(W = 24\,\mu\text{m}) = 139\,\text{mm}^{-1}$, $C_{\text{filling}}(W = 75\,\mu\text{m}) = 41\,\text{mm}^{-1}$. **b** Simulated current density profiles as a function of x for the two resonances highlighted with the arrows in panel (a). The low-frequency resonance stems from an unscreened plasmon with wavelength $\lambda \approx \frac{2}{3}W_2$, while the higher frequency resonance arises from an unscreened plasmon that forms in the transmission line gap with $\lambda \approx \frac{2}{5}W_2$.

weights. Notably, plasmonic resonances persist even at low Drude weights, such as $D_{\text{Drude}} = 63\,\mu\text{S} \times \text{THz} \times \text{rad}$. As shown in the bottom panels of the figure, these resonances overlap in frequency, giving rise to a phantom-Drude response for the chosen sample geometry.

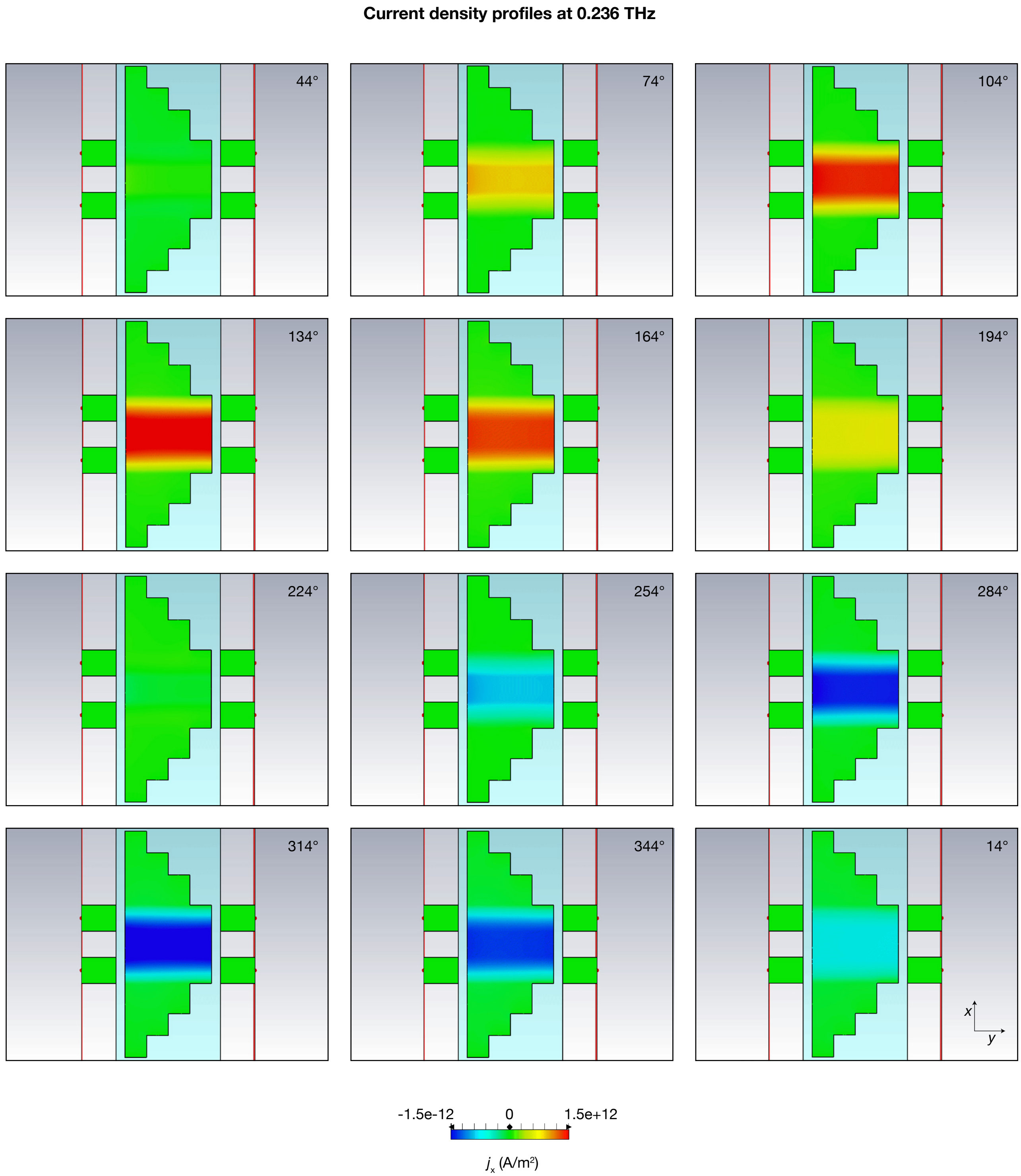

FIG. 8: **Current density distributions of the irregularly shaped device analyzed in Fig. 3 at** $0.236\,\mathrm{THz}$**, shown at different phases:** Full 3D electromagnetic simulations of the current density along the x-direction, perpendicular to the THz pulse propagation, at the frequency of the lowest-frequency plasmonic mode. In this mode, the current density in the W_0 regions remains near zero at all phases, resulting in minimal inhomogeneous broadening of the lowest-frequency resonance of the irregularly shaped sample.

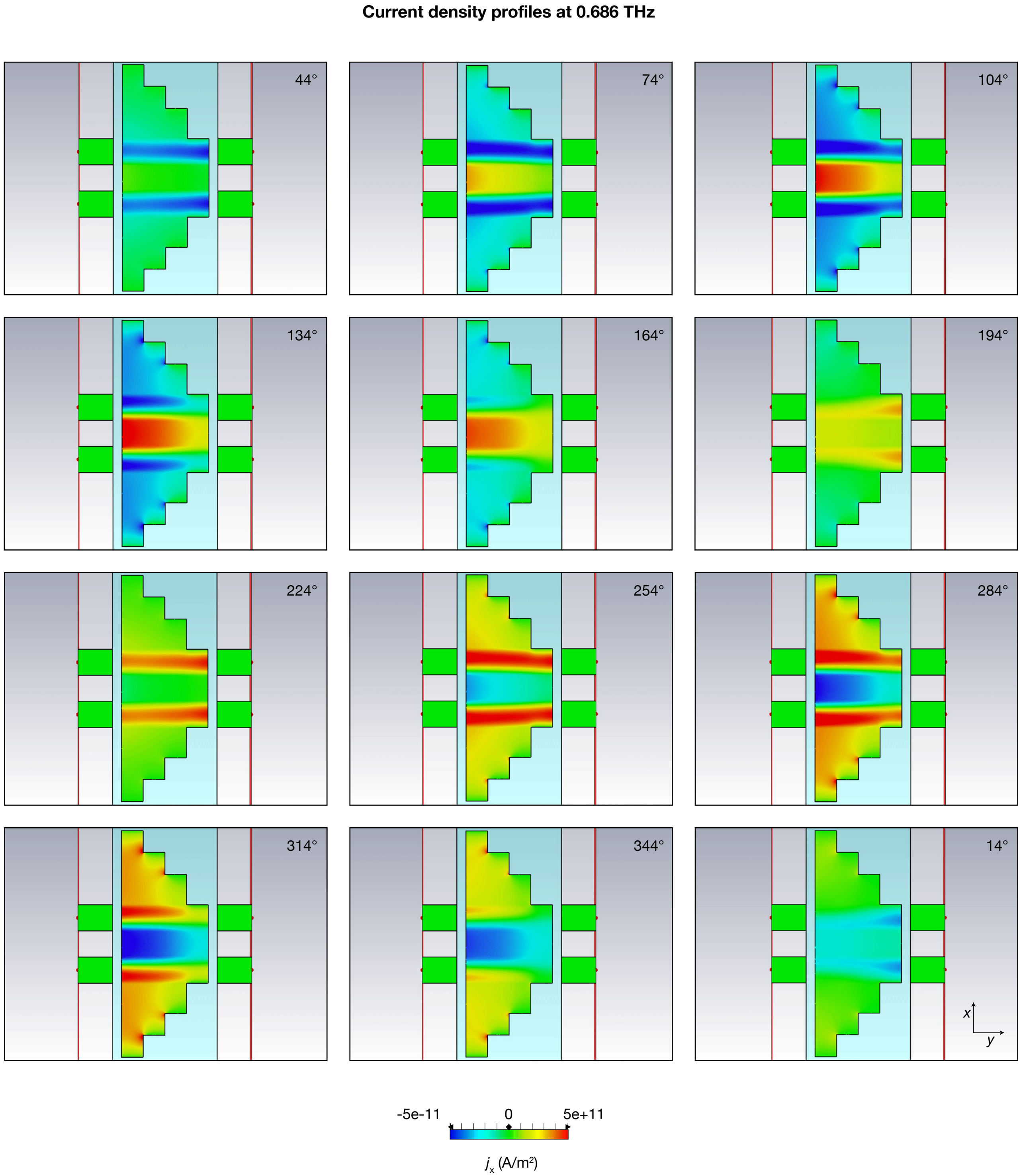

FIG. 9: **Current density distributions of the irregularly shaped device analyzed in Fig. 3 at** 0.686 THz**, shown at different phases:** Full 3D electromagnetic simulations of the current density along the x-direction, perpendicular to the THz pulse propagation, at the frequency of the second-lowest-frequency plasmonic mode. In this mode, the current density in the W_0 regions becomes comparable to that in the W_1 and W_2 regions. Since the unscreened plasmons in the W_0 regions have different wavelengths, and therefore resonance frequencies that depend on W_0, the second-lowest-frequency resonance of the irregularly shaped sample exhibits pronounced inhomogeneous broadening (see Fig. 3).

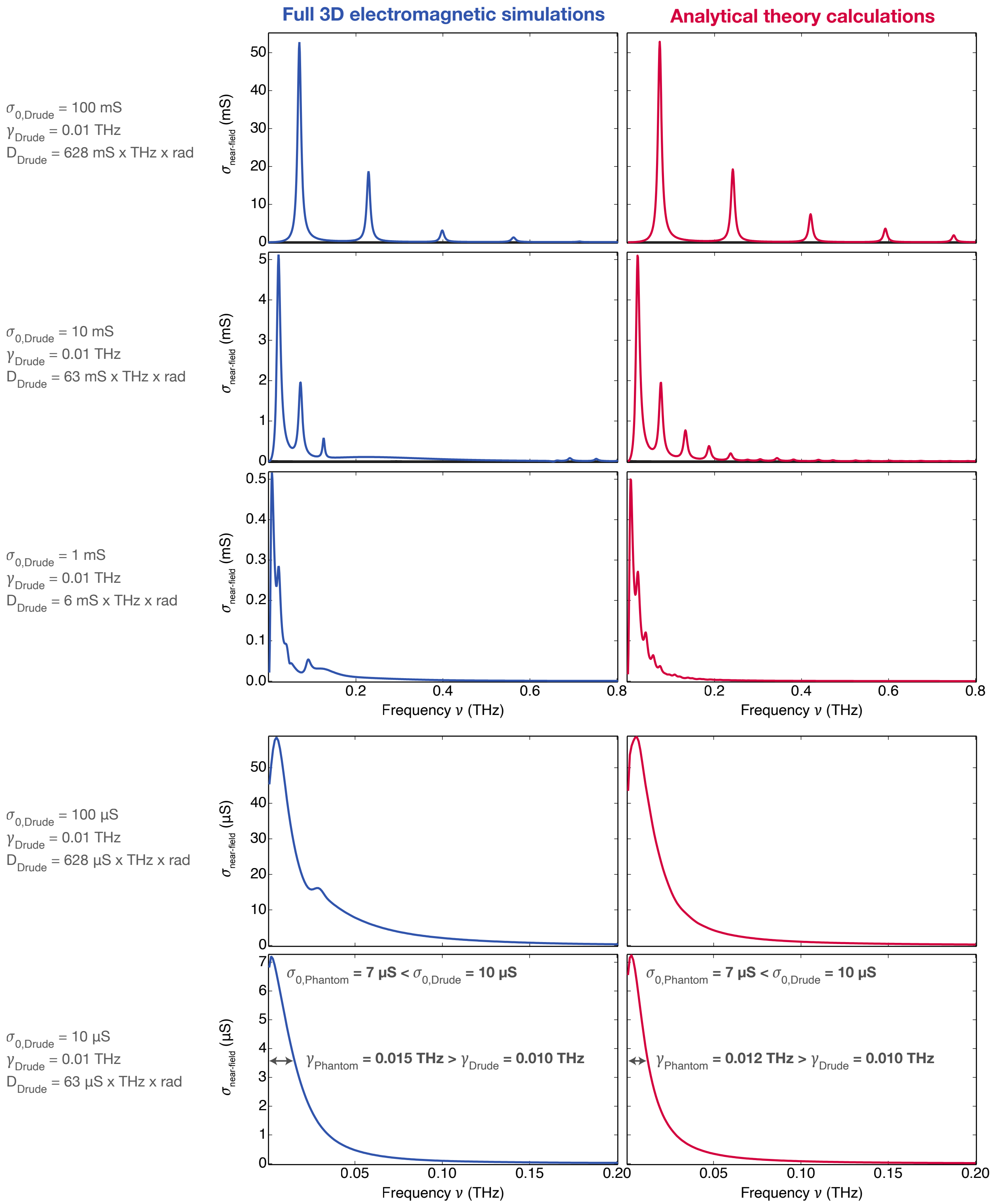

FIG. 10: **Full 3D electromagnetic simulations and analytical theory calculations of a sample with varying Drude weight:** A conductive layer with varying Drude weight $D_{\text{Drude}} = \sigma_0 \times \gamma$ and a fixed scattering rate $\gamma = 0.01\,\text{THz}$ is embedded in a heterostructure with $d_{\text{hBN}} = 100\,\text{nm}$, $W_0 = 0$ and $W_1 = W_2 = 10\,\mu\text{m}$. The system is analyzed using both full 3D electromagnetic simulations and analytical theory calculations. At the lowest simulated Drude weight, $D_{\text{Drude}} = 63\,\mu\text{S} \times \text{THz} \times \text{rad}$, the plasmonic resonances are shifted to sufficiently low frequencies that they overlap, resulting in a superposition that manifests as a Phantom-Drude response.

* Corresponding author: gukipp@phys.ethz.ch
† Corresponding author: jm5382@columbia.edu

[1] Kipp, G. et al. Cavity electrodynamics of van der waals heterostructures. Nature Physics **21**, 1926–1933 (2025). URL https://doi.org/10.1038/s41567-025-03064-8.
[2] Michael, M. H. et al. Resolving self-cavity effects in two-dimensional quantum materials. arXiv:2505.12799 (2025). URL https://arxiv.org/abs/2505.12799.
[3] Potts, A. M. et al. Finite-momentum cooper plasmons in superconducting terahertz microcavities. arXiv:2601.10692 (2026). URL https://arxiv.org/abs/2601.10692.
[4] CST. CST microwave studio advanced topics. Tech. Rep., CST-Computer Simulation Technology (2002).

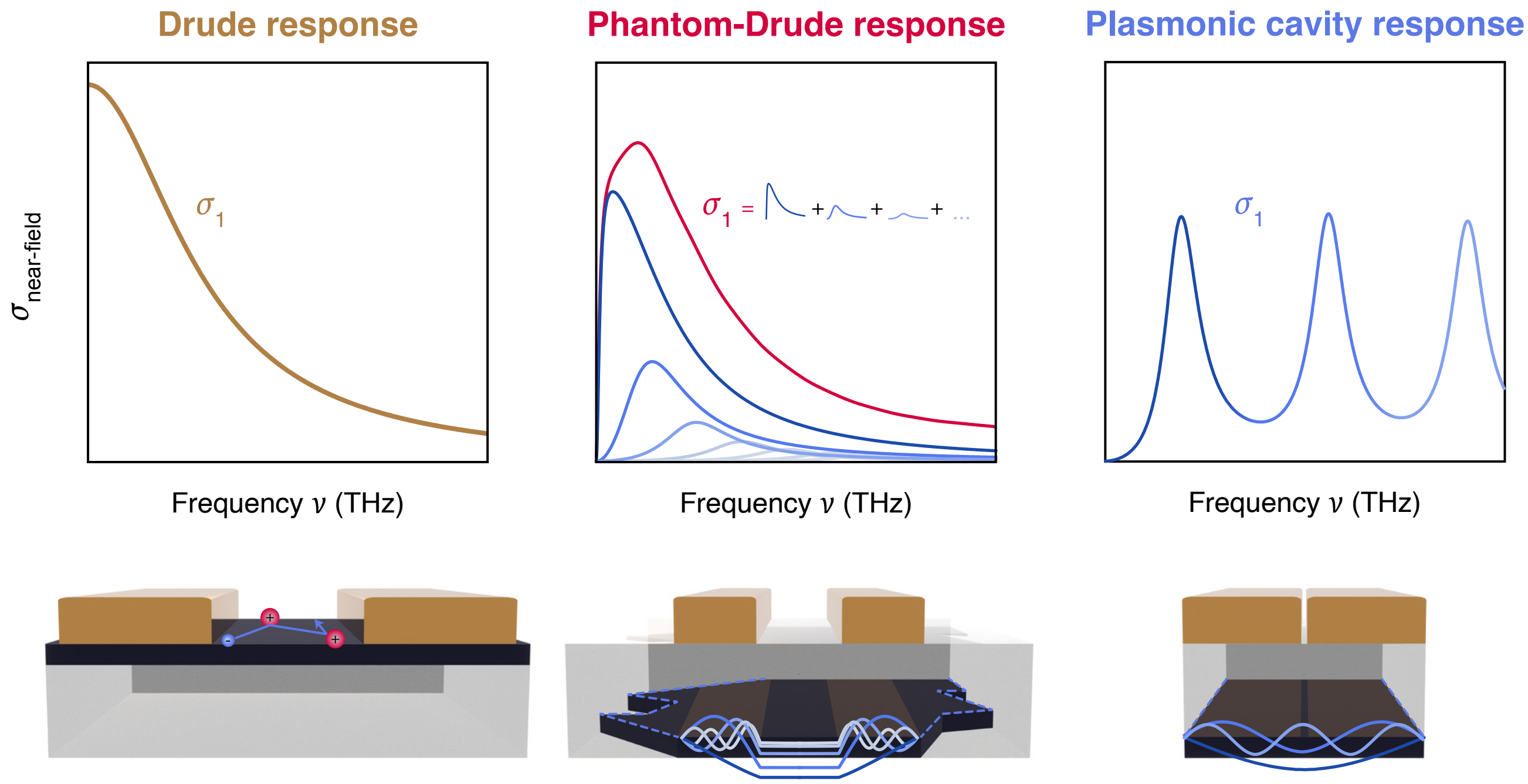

For Table of Contents Use Only: Response regimes in on-chip THz spectroscopy; Gunda Kipp, Marios H. Michael, Alexander M. Potts, Dorothee Herrmann, Toru Matsuyama, Guido Meier, Mathhew W. Day, Hope M. Bretscher, James W. McIver; This article systematically investigates how sample dimensions, transmission line geometry, material shape, and gate properties determine whether on-chip THz spectroscopy probes a genuine Drude response, a Phantom-Drude response, or plasmonic cavity electrodynamics.

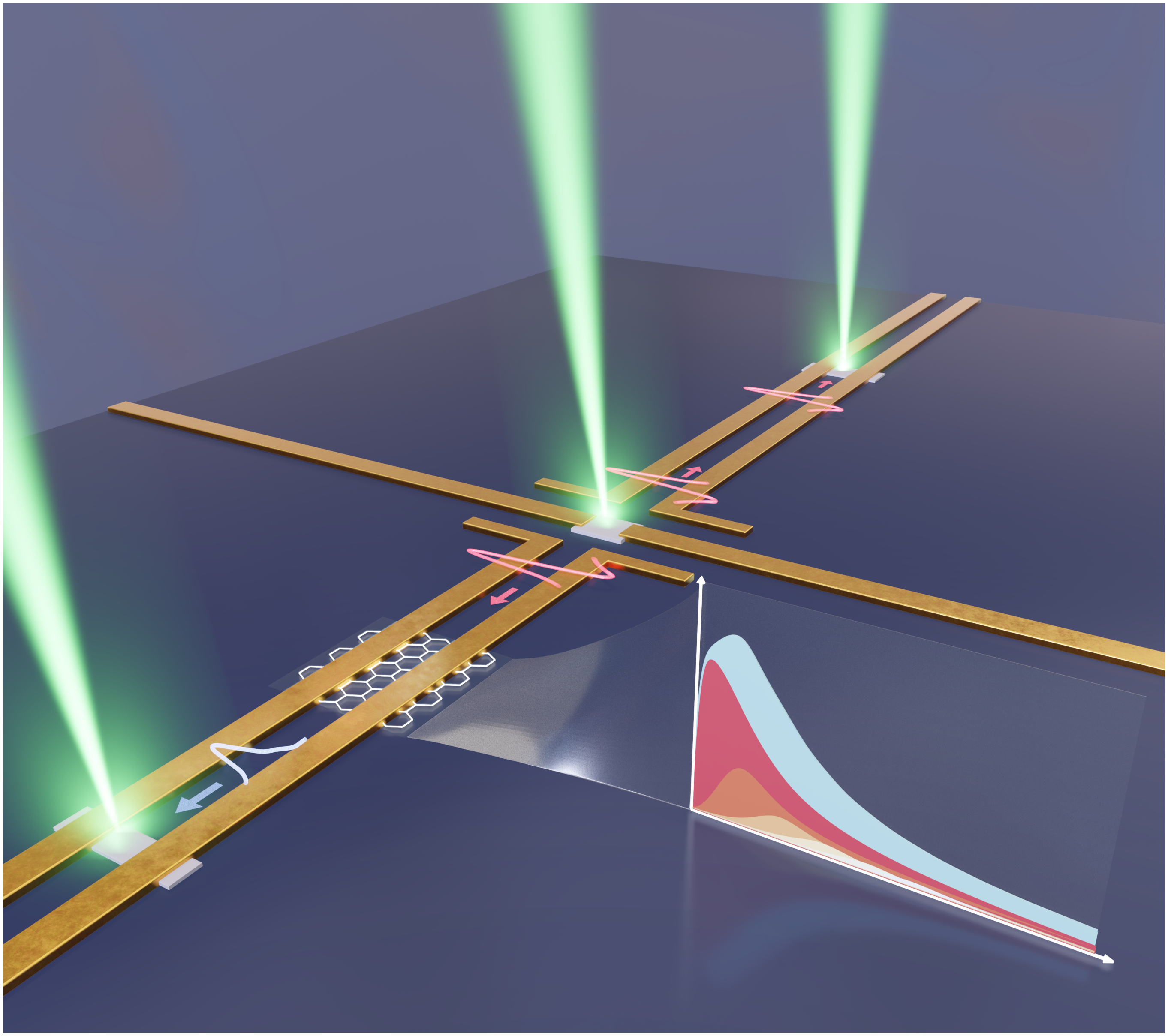

Cover art suggestion: Response regimes in on-chip THz spectroscopy; This article systematically investigates how sample dimensions, transmission line geometry, material shape, and gate properties determine whether on-chip THz spectroscopy probes a genuine Drude response, a Phantom-Drude response, or plasmonic cavity electrodynamics.
